# Stable *N*,*N’*‐Diarylated Dihydrodiazaacene Radical Cations

**DOI:** 10.1002/chem.202004548

**Published:** 2020-12-30

**Authors:** Gaozhan Xie, N. Maximilian Bojanowski, Victor Brosius, Thomas Wiesner, Frank Rominger, Jan Freudenberg, Uwe H. F. Bunz

**Affiliations:** ^1^ Organisch-Chemisches Institut Ruprecht-Karls Universität Heidelberg Im Neuenheimer Feld 270 69120 Heidelberg Germany

**Keywords:** DFT calculations, *N*,*N’*-diaryldiazaacenes, radical cation, single crystal structure, UV/Vis spectroscopy

## Abstract

Three stable *N,N’*‐diarylated dihydroazaacene radical cations were prepared by oxidation of neutral *N,N’*‐diarylated dihydroazaacenes synthesized via palladium‐catalyzed Buchwald‐Hartwig aminations of aryl iodides with *N,N’*‐dihydroazaacenes. Both neutral as well as oxidized species were investigated via UV‐vis spectroscopy, single crystal analysis, and DFT calculations. All the radical cations are surprisingly stable—their absorption spectra in dichloromethane remain unchanged in ambient conditions for at least 24 hours.

## Graphical abstract

**Oxidation of** ***N***,***N’*** **‐diarylated dihydroazaacenes** furnishes three stable dihydroazaacene radical cations characterized by means of UV‐vis spectroscopy, single crystal analysis, and DFT calculations.

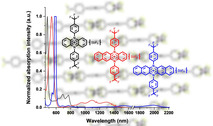

Acene‐based radicals and radical ions^[1]^ are open‐shell species with particular electronic, magnetic, and optical properties; they have potential applications in spintronics,^[2]^ organic electronics,^[3]^ organic superconductors,^[4]^ and energy storage devices.^[5]^ Prerequisite for their application is stability, since they are fairly reactive and easily form closed‐shell compounds by oxidation, dimerization, and/or disproportionation.^[6]^ At present, only a limited number of stable acene‐based radicals are described.^[7]^

6,13‐Bis(triisopropylsilylethynyl)‐5,7,12,14‐tetraazapentacene (**TAP**) is an n‐type semiconductor with electron mobilities reaching up to 10 cm^2^ V^−1^ s^−1^ in organic field‐effect transistors (OFETs).^[8]^ In 2016, Bunz, Marder, and co‐workers reported the synthesis of the radical anion **TAP^−.^** (see Scheme 1, top)^[9]^ by reducing **TAP** with potassium anthracenide. **TAP^−.^** is stable in Et_2_O solution under air for several hours (h). Later, our group reported that the radical anion **Br_4_TAP^−.^** (see Scheme 1, top)^[10]^ is stable in dry Et_2_O under air for several weeks. Recently, we described the preparation and characterization of the *N*,*N’*‐diaryldiazapentacene **Quino** and its radical cation **Quino^+.^** (see Scheme 1, bottom).^[11]^ The neutral **Quino** is unstable in dichloromethane (DCM) with around 20 % absorption intensity loss after 9 h in ambient surroundings, whereas its oxidation product, **Quino^+.^** is stable; its absorption spectrum remains unchanged for at least 24 h under the same conditions. In this contribution, we exploit acene‐based radical ions, *N*,*N’*‐dihydroazaacene‐based radical cations.^[12]^

**Scheme 1:**
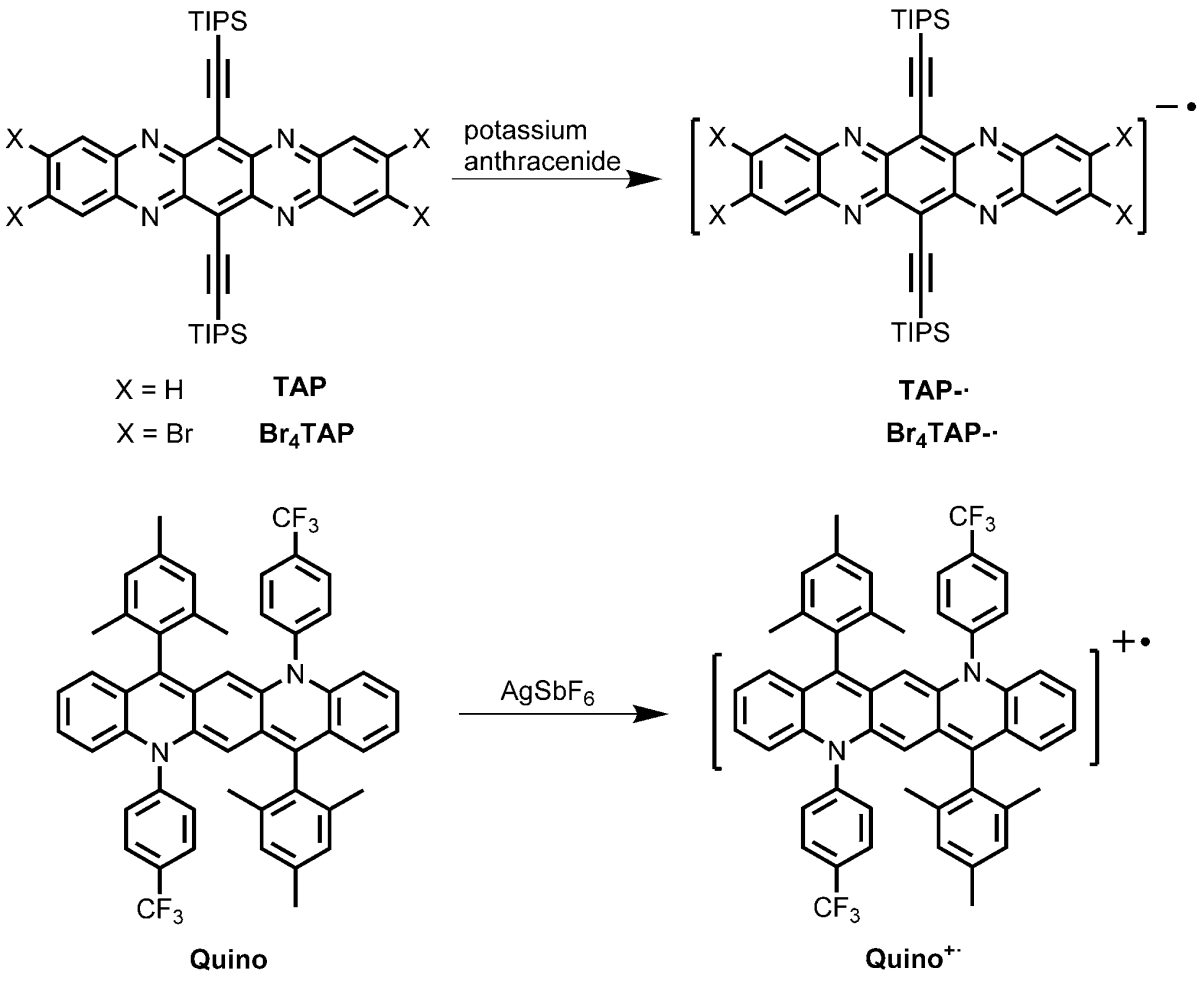
Preparation of **TAP^−.^** (top),^9^ **Br_4_TAP^−.^** (top),^10^ and **Quino^+.^** (bottom).^[11]^

**1**, **2**, and **3** were prepared by Buchwald‐Hartwig amination of 4‐iodotrifluoromethylbenzene and *N*,*N’*‐dihydroazaacenes employing a palladium catalyst under N_2_ atmosphere (Scheme 2). Yields range from 56–96 %. The electron‐withdrawing ‐CF_3_ groups retard oxidation of the electron‐rich *N*,*N’*‐dihydroazaacenes during column chromatography. Treating **1**, **2**, and **3** with one equivalent NO^+^PF_6_^−^ respectively furnished **1^+.^**, **2^+.^**, and **3^+.^** in 92–95 %, while further oxidation of the three radical cations by NO^+^PF_6_^−^ or SbCl_5_ was not possible (see Scheme S1 in the Supporting Information (SI)), although their dications were observed via cyclic voltammetry (CV, vide infra).

**Scheme 2:**
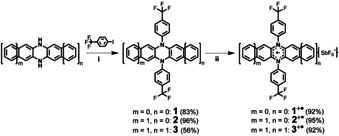
Synthetic routes to *N*,*N’*‐diarylated dihydrodiazaacenes (**DDA**s) and their corresponding radical cations (**DDA**s^+⋅^). Conditions: (i) RuPhos Pd G1, RuPhos, *t*BuOK, toluene, 110 °C, 48 h; (ii) NO^+^SbF_6_^−^, DCM, room temperature, 12 h.

Figure 1 a–c shows the EPR spectra of **DDA**s^**+**^ **^.^** in DCM. **1^+.^** and **3^+.^** display intense doublets with g‐values of 2.0022 and 2.0021. **2^+.^** exhibits a defined triplet spectrum with a g‐value of 2.0019.

**Figure 1:**
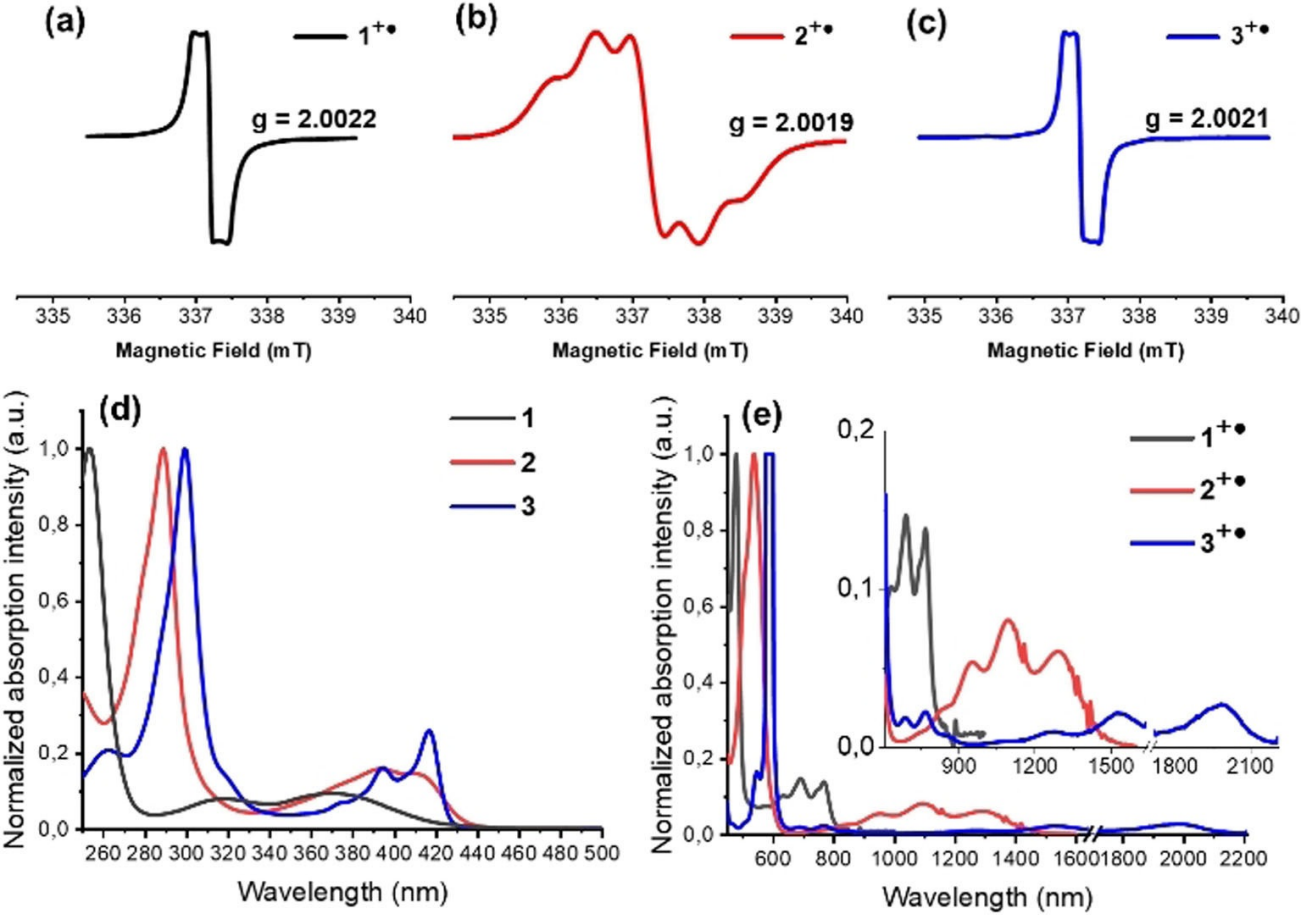
Top: electron paramagnetic resonance (EPR) spectra of (a) **1^+.^**, (b) **2^+.^**, and (c) **3^+.^** recorded in DCM under ambient conditions. See Figure S1, SI, for simulations. Bottom: normalized absorption spectra of (d) **DDA**s and (e) their radical cations measured in DCM. (Note: The characteristic extinction (from 1200 nm to 2200 nm) of **3^+.^** is extremely weak, thus a high concentration solution was measured to obtain a well‐resolved absorption spectrum without disturbing noise. However, the intensity of the absorption peak at around 590 nm was beyond measurement range under these conditions. As a consequence of detector saturation, a plateau rather than a peak is observed at around 590 nm. See Figure S2 in the SI for the dilute spectrum.).

UV‐vis absorption spectra of **DDA**s and their radical cations in DCM were measured, and TD‐DFT calculations of the vertical excited states give insight into the electronic transitions. Compound **1** displays the most hypsochromic absorption maximum (*λ*_max_) at 369 nm (see Figure 1 d) in comparison to π‐extended **2** and **3** (*λ*_max_=409 and 417 nm, respectively; see Table 1). In contrast to **1**, the absorption spectra of **2** and **3** are quite similar in overall shape. Upon oxidation into their radical cations, bathochromic‐shifted absorption spectra are observed (see Figure 1 e) with the onset of absorption for each lying in the (N)IR regimes: **1^+.^** peaks at 635, 692, and 769 nm; the absorption spectrum of **2^+.^** peaks at 955, 1095, and 1291 nm. The spectral features of **3^+.^** are the most red‐shifted with peaks at 1281, 1527, and 1986 nm. The absorption differences of the radical cation species indicate that the length of azaacene backbones dictates their spectroscopic properties. The results are consistent with the TD‐DFT calculations (see Figure S5 and Table S1 in the SI). The simulated absorption spectra of **1^+.^**, **2^+.^**, and **3^+.^** display their *λ*_max_ at 673, 1166, and 1839 nm, respectively, which are mainly contributed by the HOMO (β)→LUMO (β) transitions.

**Table 1:** Photophysical, experimental and calculated electrochemical properties of **DDA**s and their radical cations.

Comp.	λ_abs_^[a]^ (nm)	*E*_ox_^[b]^ (V)	*E*_red_^[b]^ (V)	gap^[c]^ (eV)
**1**	318, 369	0.06, 0.83	–	–
**2**	394, 409	0.51, 1.18	–	–
**3**	395, 417	0.26, 0.83	–	–
**1^+.^**	635, 692, 769	0.14	−0.63	0.77
**2^+.^**	955, 1095, 1291	0.21	−0.46	0.67
**3^+.^**	1281, 1527, 1986	0.26	−0.34	0.60

[a] Absorption peaks in DCM. [b] Oxidation and reduction potentials measured in cyclic voltammograms (CVs) using a glassy carbon working electrode, a platinum wire auxiliary electrode, a silver wire reference electrode in degassed 0.1 m NBu_4_PF_6_ DCM solution, and ferrocene/ferrocenium as the reference redox system and internal standard (−4.8 eV).^[13]^ [c] *E*_gap_=*E*_ox1_−*E*_red_.

In addition, Figure S3 (see SI) shows the evolution of absorption intensity of the radical cations as a function of time in DCM. All of the three radical cations are stable—their absorption spectra remain unchanged under ambient conditions for at least 24 h.

All dihydroacenes exhibit two reversible oxidation waves in their cyclic voltammograms (CVs, see Figure S7, SI), attributed to the formation of their radical cations and dications. **1** and **3** monooxidize readily (first oxidation potentials at 0.06 V and 0.26 V). **2** is the least easily oxidized (first and second oxidation potentials at 0.51 V and 1.18 V, versus Fc/Fc^+^). Driving force is the generation of an aromatic acene backbone. We do not observe any reduction events of the neutral specimens, due to the electron‐rich and anti‐aromatic 4n π‐electron *N*,*N’*‐dihydroazacene nuclei. The CVs of the radical cation species display both a reversible oxidation and a reversible reduction wave. From **1^+.^** to **3^+.^**, the oxidation potentials increase from 0.14 V to 0.26 V, while the reduction potentials decrease from 0.63 V to 0.34 V. The radical cations become more difficult to oxidize and easier to reduce as a consequence of extension of the π‐conjugated systems.

NICS(1)_zz_ calculations^[14]^ were performed to study the aromaticity of the neutral compounds and their radical cations. The NICS‐values of the inner *N*,*N’*‐dihydropyrazine (see Figure 2) are positive, with their values surpassing +19.8, indicating local antiaromaticity.^[15]^ This antiaromaticity decreases from **1** to **3**, which might be ascribed to the difference in bond orders of the fused bond between the dihydropyrazine core and the outer arene groups (benzene, naphthalene and a combination of both).^[16]^ The NICS‐values of the peripheral arene rings are negative, they are aromatic. Upon oxidation, the aromaticity of the central *N*,*N’*‐dihydropyrazine rings increases dramatically, and both the *N*,*N’*‐dihydropyrazine rings of **2^+.^** and **3^+.^** now are aromatic with the NICS‐values decreasing to −2.02 and −4.55 respectively.

**Figure 2:**
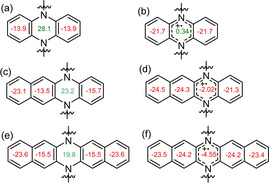
NICS(1)_zz_ values of a) **1**; b) **1^+.^**; c) **2**; d) **2^+.^**; e) **3**; f) **3^+.^** calculated with Gaussian 16 at B3LYP/6–311++G**//DFT/B3LYP/6–311+G** level of theory.^[17]^

The frontier molecular orbital distributions of **DDA**s and **DDA**s^**+**^ **^.^** are depicted in Figure S6 in the SI. For the neutral species, their highest occupied molecular orbitals (HOMOs) are distributed over the whole backbone, while the lowest occupied molecular orbitals (LUMOs) are mainly located on the electron‐withdrawing *para*‐trifluoroaryl side groups, differing from aryl‐substituted acenes such as azarubrenes,^[18]^ HOMOs and LUMOs of which are all distributed on the acene backbones. For **DDA**s^**+**^ **^.^**, their singly occupied molecular orbitals (SOMOs) are mostly distributed on the dihydrodiazaacene cores, as expected.

We cultivated single crystals of the neutral **2** and **3** for XRD analysis by slow evaporation of saturated solutions of DCM under ambient conditions. The single crystal structure of **1** was already reported.^[19]^ In addition, the single crystals of **1^+.^**, **2^+.^**, and **3^+.^** were grown successfully by slow evaporation of acetone solutions under air. For all of the six compounds, their *N*,*N’*‐(dihydro)diazaacene backbones are planar (see Figure 3 and Figure S8 in the SI) with the *para*‐trifluoroaryl side groups almost orthogonal to the azaacene backbones. Upon monooxidation, the neutral specimens display pronounced bond length alternations over the whole azaacene backbone, in accordance with DFT calculations. For the radical cation species, C−N bond lengths are shorter compared to those of the neutral species, which indicates stronger conjugation between nitrogen atoms and adjacent phenyl rings in the oxidized diazaacenes, consistent with the NICS(1)_zz_ calculations.

**Figure 3:**
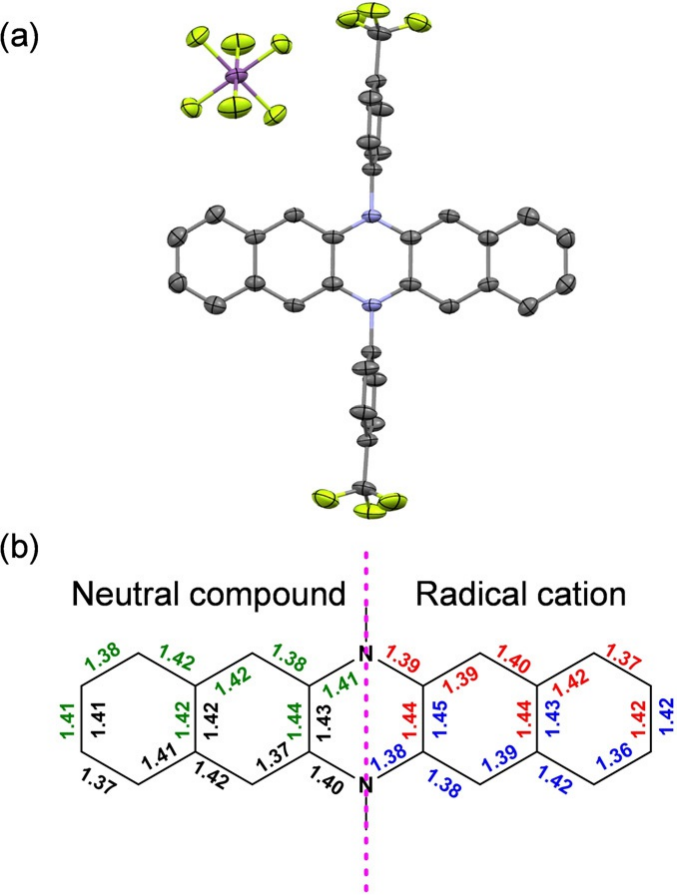
(a) Single crystal structure of **3^+.^**; (b) averaged bond lengths of all equivalent bonds (green and red denote to computational bond lengths of **3** and **3^+.^** respectively, calculated at the DFT/B3LYP/6–311+G** level of theory^13^; black: crystal structure bond lengths of **3**; blue: crystal structure bond lengths of **3^+.^**).

In conclusion, we present a practical strategy to exploit stable radical cations based on *N*,*N’*‐dihydropyrazine units. *N*,*N’*‐Diarylated dihydroazaacenes, **1**, **2**, and **3**, were synthesized through Buchwald‐Hartwig amination of aryl iodides with *N*,*N’*‐dihydroazaacenes. Upon monooxidation of the neutral compounds, the radical cations **1^+.^**, **2^+.^**, and **3^+.^** formed, stable in DCM under ambient conditions for at least 24 h. In comparison with the neutral species, the radical cation species display bathochromically‐shifted absorption spectra with a *λ*_max_ of 769, 1291, and 1986 nm, respectively. The alternation of bond lengths between the neutral compounds and their radical cations in single crystal structures as well as the NICS(1)_zz_ calculations indicate the *N*,*N’*‐dihydropyrazine rings of the radical cations to be less antiaromatic. In the future, we will prepare *N*,*N’*‐dihydrodiazaacene radical cations with longer backbones, *N*,*N’*‐dihydrodiazahexacene and *N*,*N’*‐dihydrodiazapentacene radical cations. Further attempts to electrochemically oxidize the *N*,*N’*‐dihydroazaacenes to their fully aromatic dications are under way.

## Experimental Section

The radical cations were synthesized in a glove box under N_2_. 5,10‐Di(4‐trifluoromethylphenyl)‐5,10‐dihydrophenazine (**1**),^[19]^ 5,12‐dihydrobenzo‐[*b*]‐phenazine,^[20]^ and 6,13‐dihydrodibenzo‐[*b,i*]phenazine^[21]^ were synthesized according to reported literatures.

**General procedure (GP1)**: To a stirring solution of *N*,*N’*‐disubstituted dihydrophenazine derivative (50.0 mg, 1.00 equivalent (equiv.)) in 15 mL DCM was added dropwise NO^+^SbF_6_^−^ (1.05 equiv.) in 1.5 mL CH_3_CN and the reaction mixture was stirred for 12 h at room temperature (r.t.). After that, the mixture was filtered through filter paper and the solvent was removed under reduced pressure to give the corresponding radical cations.

**General procedure for Buchwald‐Hartwig amination (GP2)**: The dihydrophenazine derivative (1.00 equiv.), 1‐iodo‐4‐(trifluoromethyl)benzene (4.00 equiv.), RuPhos Pd G1 (0.05 equiv.), *t*BuOK (6.00 equiv.), and RuPhos (0.10 equiv.) were added into a flask under N_2_. After exposing the flask to a flow of N_2_ for 30 min to obtain an inert atmosphere, toluene was added. The mixture was allowed to heat to 100 °C and stirred for 36 h. Thereafter, the solution was filtered through a silica pad with ethyl acetate (EE), and then concentrated under reduced pressure before further purification steps were undertaken.

**1^+⋅^**: **1** (50.0 mg, 106 μmol) and NO^+^SbF_6_^−^ (29.7 mg, 111 μmol,) were used in combination with **GP1. 1^+.^** was obtained as a deep green solid. Yield: 69.1 mg, 97.8 μmol, 92 %. Single crystals suitable for X‐ray crystallography were grown from saturated acetone solution. IR: *ṽ*=3086, 2920, 2851, 1604, 1559, 1466, 1319, 1060, 649 cm^−1^. MS (MALDI) *m*/*z*: [M]^+^: calcd for C_26_H_16_N_2_F_6_^+⋅^: 470.121; found 470.172; correct isotope distribution; elemental analysis calcd for C_30_H_18_N_2_F_6_^+⋅^: C: 44.22 %, H: 2.28 %, N: 3.97 %, found: C: 44.16 %, H: 2.50 %, N: 4.22 %.

**5,12‐Di(4‐trifluoromethylphenyl)‐5,12‐dihydrobenzo[*b*]phenazine (2)**: 5,12‐Dihydrobenzo[*b*]phenazine (500 mg, 2.15 mmol), 1‐iodo‐4‐(trifluoromethyl)benzene (2.34 g, 8.61 mmol), RuPhos Pd G1 (88.0 mg, 108 μmol), *t*BuOK (1.45 g, 12.9 mmol), and RuPhos (100 mg, 215 μmol) were used in combination with **GP2** in toluene (20 mL). After purification via column chromatography (silica gel, DCM) and evaporation of the solvent in vacuo, the crude product was further washed with petroleum (PE) and a faint yellow solid was obtained. Yield: 1.08 g, 2.07 mmol, 96 %. Melting point (mp): 305 °C. ^1^H NMR (CD_2_Cl_2_, 600 MHz, 295 K): *δ*=8.05–7.92 (m, 4 H), 7.66–7.60 (m, 4 H), 7.09–7.03 (m, 2 H), 7.00–6.95 (m, 2 H), 6.42–6.33 (m, 2 H), 5.85–5.80 (s, 2 H), 5.75–5.67 (m, 2 H) ppm. ^13^C{^1^H} NMR (CD_2_Cl_2_, 151 MHz, 295 K): *δ*=143.7, 136.6, 134.7, 132.5, 131.0, 129.3, 126.0, 125.5, 124.7, 123.7, 121.6, 113.4, 108.0 ppm. IR: *ṽ*=3059, 1475, 1332, 1120, 1060, 843, 732 cm^−1^. MS (MALDI) *m*/*z*: [M]^+^: calcd for C_30_H_18_N_2_F_6_: 520.478; found 520.098; correct isotope distribution.

**2^+⋅^**: **2** (50.0 mg, 96.1 μmol) and NO^+^SbF_6_^−^ (26.8 mg, 101 μmol) were used in combination with **GP1. 2^+.^** was obtained as a dark red solid. Yield: 69.0 mg, 91.3 μmol, 95 %. Single crystals suitable for X‐ray crystallography were grown from saturated acetone solution. IR: *ṽ*=3063, 2980, 2915, 1600, 1435, 1323, 1065, 645 cm^−1^. MS (MALDI) *m*/*z*: [M]^+^: calcd for C_30_H_18_N_2_F_6_^+⋅^: 520.137; found 520.206; correct isotope distribution; elemental analysis calcd for C_30_H_18_N_2_F_6_^+⋅^: C: 47.65 %, H: 2.40 %, N: 3.70 %, found: C: 47.79 %, H: 2.83 %, N: 3.68 %.

**6,13‐Di(4‐trifluoromethylphenyl)‐6,13‐dihydrodibenzo[*b,i*]phenazine (3)**: 6,13‐Dihydrodibenzo[*b,i*]phenazine (200 mg, 703 μmol), 1‐iodo‐4‐(trifluoromethyl)benzene (765 mg, 2.81 mmol), RuPhos Pd G1 (28.7 mg, 35.2 μmol), *t*BuOK (474 mg, 4.22 mmol), and RuPhos (32.8 mg, 70.3 μmol) were used in combination with **GP2** in toluene (20 mL). After purification by column chromatography (silica gel, DCM) and evaporation of the solvent in vacuo, the residue was washed by PE to give a yellow solid. Yield: 226 mg, 395 μmol, 56 %. Mp: >400 °C (decomposition). ^1^H NMR (CD_2_Cl_2_, 400 MHz, 295 K): *δ*=8.10–8.01 (m, 4 H), 7.74–7.67 (m, 4 H), 7.18–7.12 (m, 4 H), 7.06–6.99 (m, 4 H), 6.02–5.97 (s, 4 H) ppm. ^13^C{^1^H} NMR (CD_2_Cl_2_, 151 MHz, 295 K): *δ*=143.7, 136.6, 134.7, 132.5, 131.0, 129.3, 126.0, 125.5, 124.7, 123.7, 121.6, 113.4, 108.0 ppm. IR: *ṽ*=3059, 1614, 1457, 1305, 1129, 1060, 843, 746 cm^−1^. MS (MALDI) *m*/*z*: [M]^+^: calcd for C_34_H_20_N_2_F_6_: 570.538; found 570.373; correct isotope distribution; elemental analysis calcd for C_34_H_20_N_2_F_6_: C: 71.58 %, H: 3.53 %, N: 4.91 %, found: C: 71.43 %, H: 3.81 %, N: 4.94 %.

**3^+⋅^**: **3** (50.0 mg, 87.6 μmol) and NO^+^SbF_6_^−^ (24.5 mg, 92.0 μmol) were used in combination with **GP1. 3^+.^** was obtained as a brown solid. Yield: 65.0 mg, 80.6 μmol, 92 %. Single crystals suitable for X‐ray crystallography were grown from saturated acetone solution. IR: *ṽ*=3054, 2925, 2856, 2754, 1591, 1314, 1055, 645 cm^−1^. MS (MALDI) *m*/*z*: [M]^+^: calcd for C_34_H_20_N_2_F_6_^+⋅^: 570.153; found 570.243; correct isotope distribution.

Deposition number(s) 2018606 (**1^+.^**), 2018607 (**2**), 2018608 (**2^+.^**), 2018609 (**3**), and 2018610 (**3^+.^**) contain the supplementary crystallographic data for this paper. These data are provided free of charge by the joint Cambridge Crystallographic Data Centre and Fachinformationszentrum Karlsruhe Access Structures service www.ccdc.cam.ac.uk/structures.

## Conflict of interest

The authors declare no conflict of interest.

## Supporting information

As a service to our authors and readers, this journal provides supporting information supplied by the authors. Such materials are peer reviewed and may be re‐organized for online delivery, but are not copy‐edited or typeset. Technical support issues arising from supporting information (other than missing files) should be addressed to the authors.

Supplementary
