# Supplementary material for: Stable N,N’‐Diarylated Dihydrodiazaacene Radical Cations
Source: Chemistry. 2020 Dec 30;27(6):1976–80. doi: 10.1002/chem.202004548 (PMC7898594; doi:10.1002/chem.202004548)
Supplement: Supplementary file 1 — Supplementary [file CHEM-27-1976-s001.pdf]

Chemistry–A European Journal

Supporting Information

Stable *N*,*N*′-Diarylated Dihydrodiazaacene Radical Cations

Gaozhan Xie, N. Maximilian Bojanowski, Victor Brosius, Thomas Wiesner, Frank Rominger, Jan Freudenberg,* and Uwe H. F. Bunz*[a]

Index:

S1. General Remarks 02

S2. Syntheses 03

S3. Experimental and Simulated EPR Spectra 04

S4. Absorption Spectra 05

S5. Theoretical Studies 07

S6. Electrochemistry 09

S7. NMR Spectra 10

S8. Mass Spectra 18

S9. Infrared Spectra 23

S10. Crystal Structures 28

S11. Cartesian Coordinates of Computational Molecules 34

S12. References 40

S1. General Remarks

Thin layer chromatography (TLC) was carried out on Polygram SILG/UV254 plates from Macherey, Nagel&Co.KG (Düren, Germany). Plates were examined under ultraviolet irradiation (254 nm and 365 nm). Melting points (Mp) were determined in open glass capillaries with a Melting Point Apparatus MELTEMP (Electrothermal, Rochford, UK). NMR spectra (^{1}H, ^{13}C) were recorded on Bruker Avance III 400 or Bruker Avance III 600 spectrometers. Chemical shifts (δ) are given in parts per million (ppm) relative to internal solvent signals.[1] The following abbreviations describe the signal multiplicities: s = single m = multiplet. IR and absorption spectra were recorded on a JASCO FT/IR-4100 and a Jasco UV-Vis V-670 respectively. High resolution mass spectra (HR-MS) were obtained from matrix-assisted laser desorption/ionization (MALDI) on a Bruker ApexQe hybrid 9.4 TFT-ICR-MS. X-ray single-crystal structure analyses were measured on a Bruker Smart APEX-II Quazar Area Detector diffractometer by Dr. Frank Rominger (Heidelberg University).

S2. Syntheses

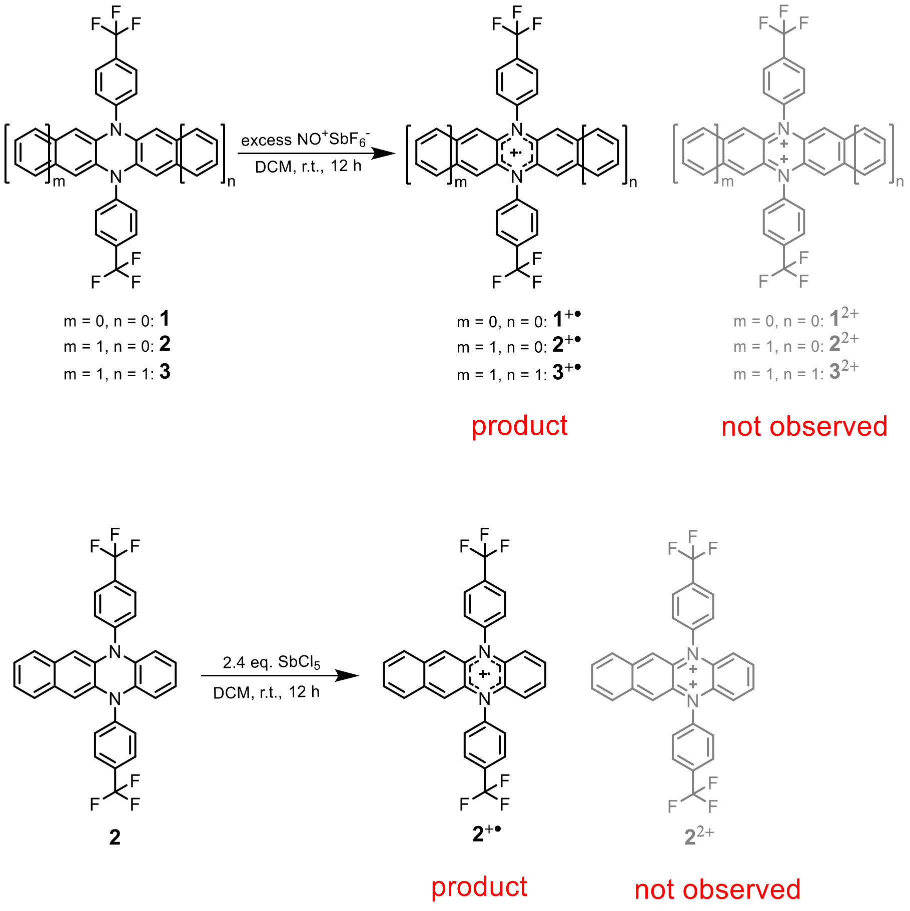

Scheme S1. Failed synthetic routes to *N,N'*-diarylated dihydrodiazaacene dications using excess equivalents of $NO^+PF_6^-$ or $SbCl_5$. Only the corresponding radical monocations were obtained.

S3. Experimental and Simulated EPR Spectra

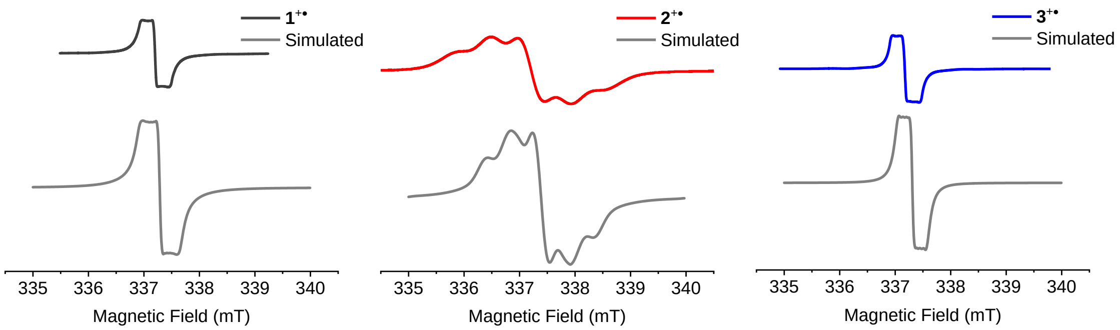

Figure S1. Comparision between experimental (colored) and simulated (grey) EPR spectra of **1**-**3** radical cations. The data of the EPR spectra given were fitted with the EasySpin simulation package[2] as isoptropic spectra (function: garlic) assuming no coupling to N and the closest protons (in case of $\mathbf{1}^{+\bullet}$ 8 Protons, $\mathbf{2}^{+\bullet}$ 6 Protons and $\mathbf{3}^{+\bullet}$ the inner four protons). The differences in the spectra as such are a result of the different number of nuclei coupling to the unpaired electron: For all compounds, only the neighboring aromatic protons have to be taken into account. If the molecular backbone increases in size, this does not have any influence on the localization of the charge.

S4. Absorption Spectra

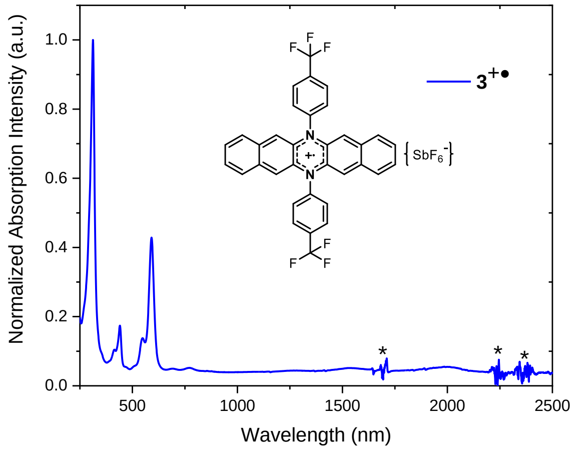

Figure S2. Normalized absorption spectrum of $3^{+\cdot}$ in dilute solution of DCM. Artifacts (*) in the spectrum are caused by our instrumentation.

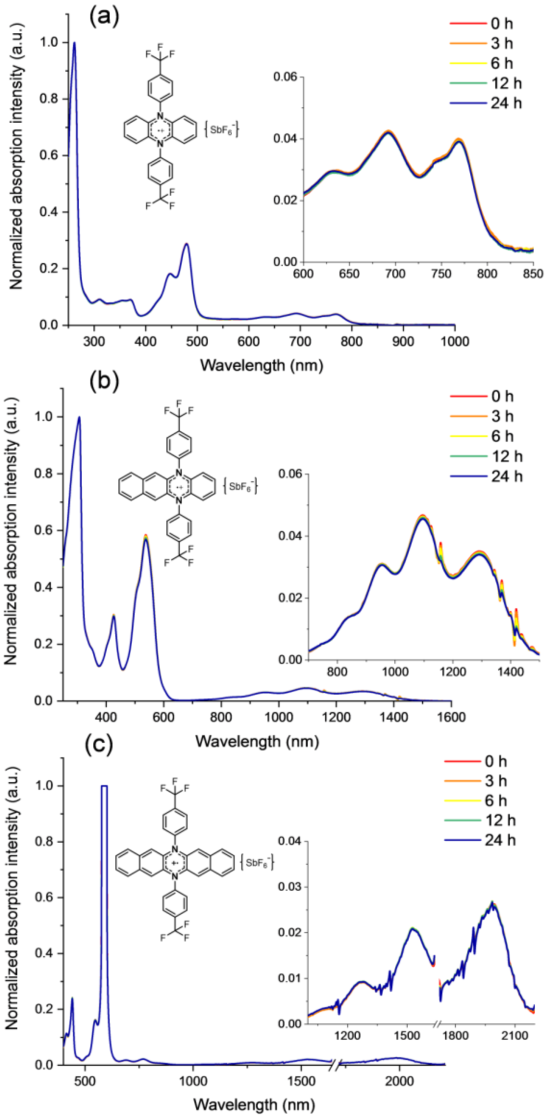

Figure S3. Normalized time-dependent evolution of absorption spectra of a) **1**$^{+\cdot}$; b) **2**$^{+\cdot}$; c) **3**$^{+\cdot}$ measured in DCM.

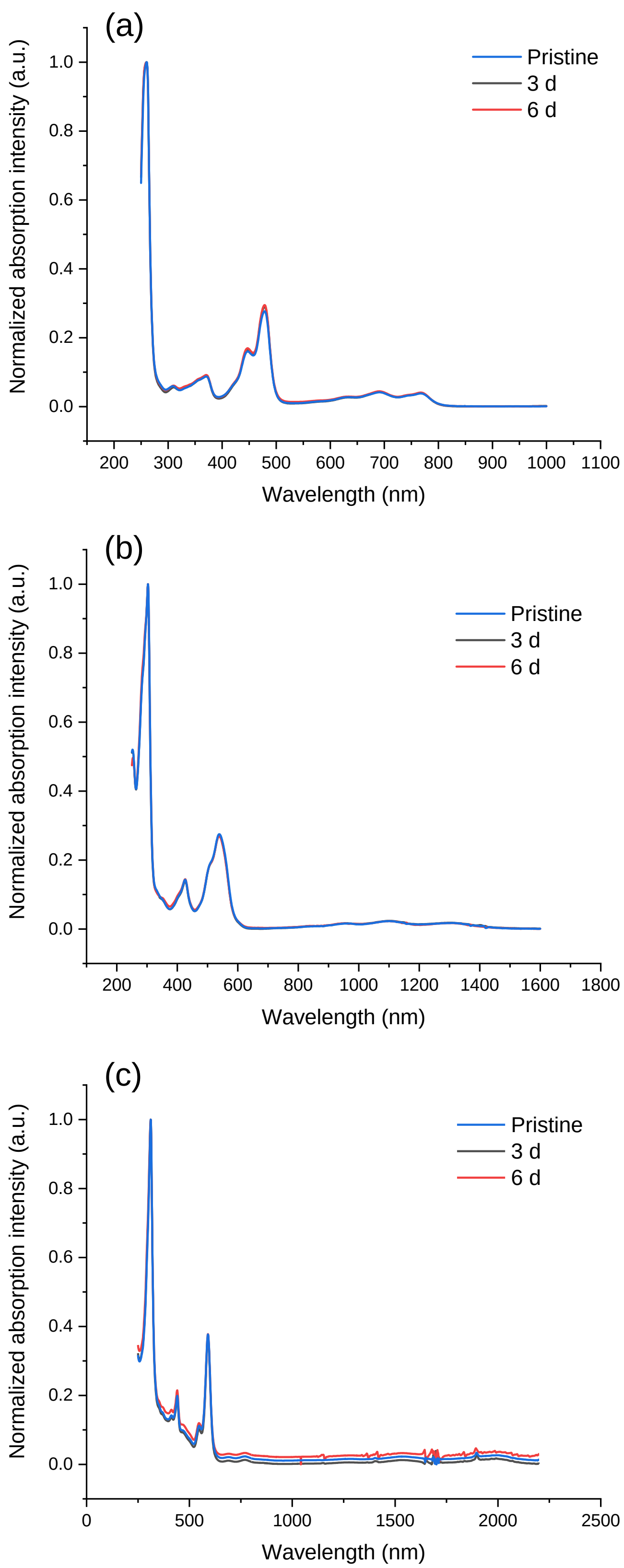

Figure S4. Normalized time-dependent evolution of absorption spectra of a) $\mathbf{1}^{+\cdot}$; b) $\mathbf{2}^{+\cdot}$; c) $\mathbf{3}^{+\cdot}$ measured in DCM.

S5. Theoretical Studies

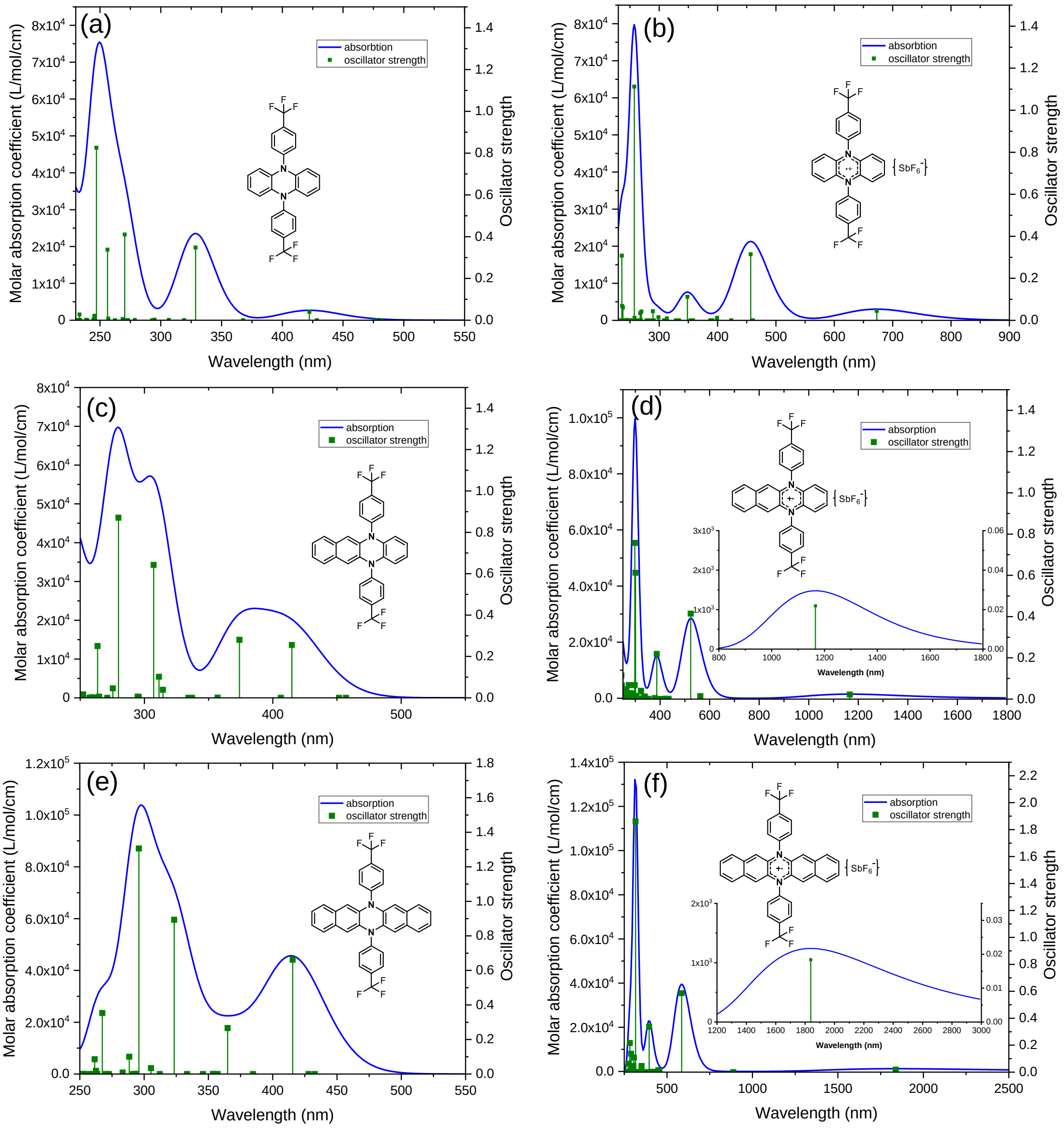

Figure S5. Simulated absorption spectra of a) **1**; b) **1**$^{+\cdot}$; c) **2**; d) **2**$^{+\cdot}$; e) **3**; f) **3**$^{+\cdot}$ calculated by Gaussian 16 at TD-DFT/B3LYP/6-311++G**//DFT/B3LYP/6-311+G**/DCM level of theory.[3] The spectra were broadened using Gaussian functions with a full-width at half maximum of 0.2 eV.

Table S1. Selected TD-DFT (Gaussian 16 B3LYP/6-311++G**//DFT/B3LYP/6-311+G**/DCM)[3] calculated energies, oscillator strengths, and compositions of major electronic transitions of **DDAs** and their radical cations.

Comp.	Abs. (nm)	Osc. Strengths (f)	Major Transition Contributions
**1**	329	0.3481	HOMO -> L+5 (69.1%)
	422	0.0398	HOMO -> L+2 (70.4%)
**2**	307	0.6425	H-1 -> L+3 (68.4%)
	374	0.2805	HOMO -> L+4 (68.9%)
	415	0.2552	HOMO -> L+2 (69.1%)
**3**	323	0.8932	H-1 -> L+3 (69.3%)
	365	0.2662	HOMO -> L+1 (69.7%)
	416	0.6623	HOMO -> L+1 (69.0%)
$\mathbf{1}^{+\cdot}$	349	0.1120	HOMO (α) -> L+3 (α) (94.3%)
	457	0.3146	H-1 (β) -> LUMO (β) (97.8%)
	673	0.0448	HOMO (β) -> LUMO (β) (99.0%)
$\mathbf{2}^{+\cdot}$	387	0.2190	HOMO (α) -> LUMO (α) (92.6%)
	524	0.4145	H-1 (β) -> LUMO (β) (98.7%)
	1166	0.0219	HOMO (β) -> LUMO (β) (99.1%)
$\mathbf{3}^{+\cdot}$	395	0.3366	HOMO (α) -> LUMO (α) (94.7%)
	586	0.5862	H-2 (β) -> LUMO (β) (99.0%)
	1839	0.0184	HOMO (β) -> LUMO (β) (99.5%)

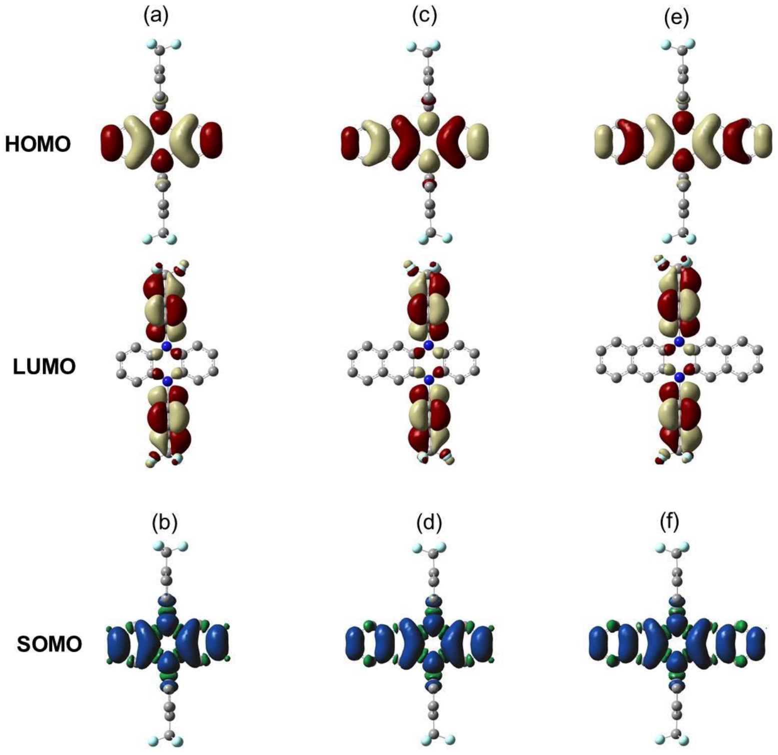

Figure S6. HOMOs and LUMOs (top) of a) **1**; c) **2**; e) **3** calculated at DFT/B3LYP/6-311++G**//DFT/B3LYP/6-311+G** level of theory. Spin-density distributions (bottom) of b) $\mathbf{1}^{+\cdot}$; d) $\mathbf{2}^{+\cdot}$; f) $\mathbf{3}^{+\cdot}$ calculated at DFT/UB3LYP/6-311++G**//DFT/B3LYP/6-311+G** level of theory.[3]

S6. Electrochemistry

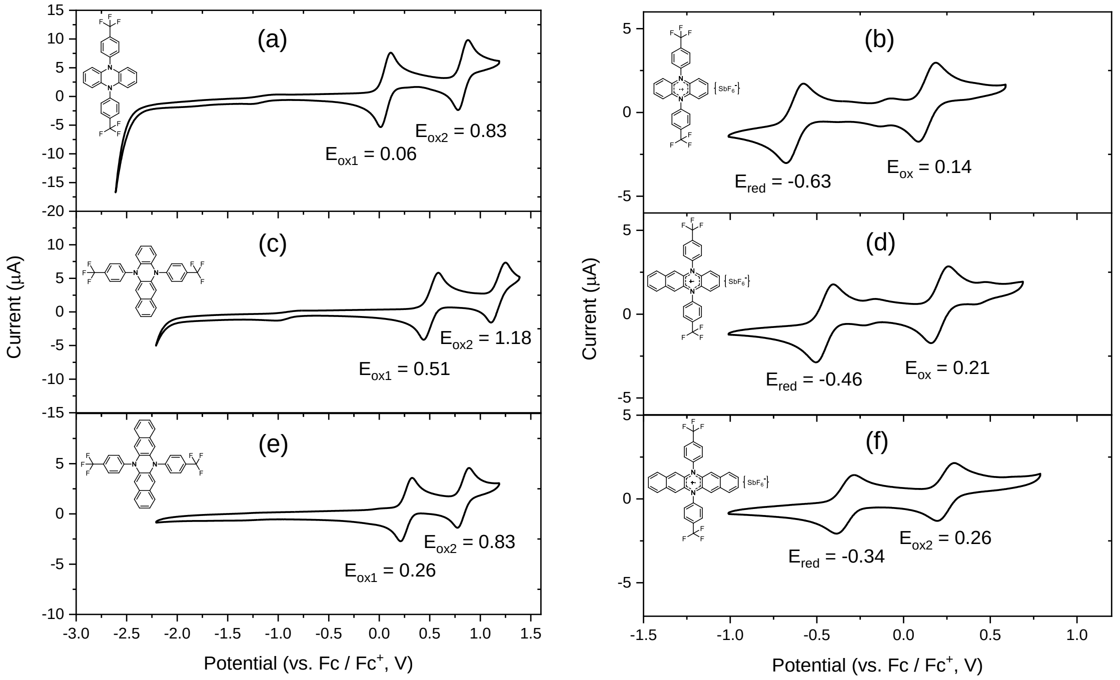

Figure S7. Cyclic voltammograms of a) **1**; b) **1**$^{+\cdot}$; c) **2**; d) **2**$^{+\cdot}$; e) **3**; f) **3**$^{+\cdot}$using a glassy carbon working electrode, a platinum/titanium wire auxiliary electrode, and a silver wire reference electrode in degassed 0.1 M NBu_4PF_6 DCM solution.[4] The electrode potential was externally calibrated by Fc/Fc^+ couple.

S7. NMR Spectroscopy

^{1}H-NMR and $^{13}C\{^{1}H\}$ NMR of **2**

Multiplets Integrals Sum	18.07	*Number of Nuclei*	18 H's

Acquisition Time (sec)	3.6351	***Comment***	GZ 475	***D***	0.1	***D1***	0.1	***DE***	12
DS	2	***Date***	07 Oct 2019 14:25:26			***Date Stamp***	07 Oct 2019 14:25:26		
File Name	\\bunz22\Mitarbeiter\Gaozhan XIE\03_analytics\01-NMR\nmr-biradical01\e191007ubgz.475\2\PDATA\1\1r							***Frequency (MHz)***	600.2438
GB	0	***INSTRUM***	<spect>	***LB***	0.3	***NS***	128	***Nucleus***	1H
Number of Transients	128	***Origin***	spect	***Original Points Count***	65536	***Owner***	ns	***PC***	1
PROBHD	<Z132808_0001 (CP QCI 600S3 H/P/C-N-D-05 Z LT)>			***PULPROG***	<zg30>	***Points Count***	65536	***Pulse Sequence***	zg30
Receiver Gain	15.35	***SF***	600.243829	***SFO1***	600.246830219145			***SI***	65536
SSB	0	***SW(cyclical) (Hz)***	18028.85	***SWH***	18028.8461538462				
Solvent	DICHLOROMETHANE-d2			***Spectrum Offset (Hz)***	2807.2170	***Spectrum Type***	standard	***Sweep Width (Hz)***	18028.57
TD	131072	***TD0***	16	***TE***	295.0004	***Temperature (degree C)***	22.000	***UNC1***	<1H>
WDW	1								

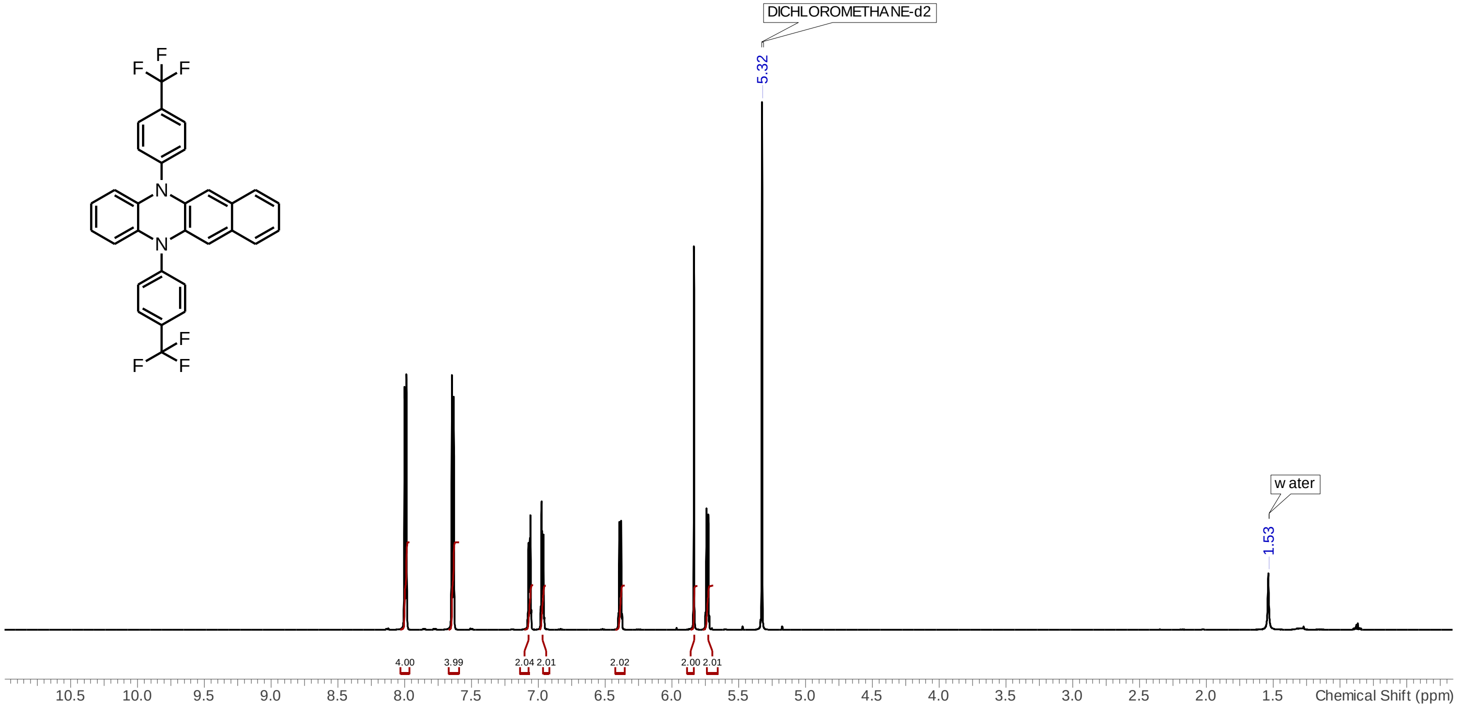

Multiplets Integrals Sum	0.00	Number of Nuclei	0 C's

Acquisition Time (sec)	1.0795	Comment	GZ 475	D	0.03	D1	2	DE	18
DS	4	Date	07 Oct 2019 14:16:15			Date Stamp	07 Oct 2019 14:16:15		
File Name	\\bunz22\Mitarbeiter\Gaozhan XIE\03_analytics\01-NMR\nmr-biradical01\e191007ubgz.475\1\PDATA\1\1r							Frequency (MHz)	150.9314
GB	0	INSTRUM	<spect>	LB	1	NS	3160	Nucleus	13C
Number of Transients	3160	Origin	spect	Original Points Count	49066	Owner	ns	PC	1.4
PROBHD	<Z132808_0001 (CP QCI 600S3 H/P/C-N-D-05 Z LT)>			PULPROG	<zgpg30>	Points Count	65536	Pulse Sequence	zgpg30
Receiver Gain	2050.00	SF	150.931431	SFO1	150.94803345741			SI	65536
SSB	0	SW(cyclical) (Hz)	45454.55	SWH	45454.5454545455				
Solvent	DICHLOROMETHANE-d2			Spectrum Offset (Hz)	16644.2480	Spectrum Type	standard	Sweep Width (Hz)	45453.85
TD	98132	TD0	512	TE	294.9991	Temperature (degree C)	21.999	UNC1	<13C>
WDW	1								

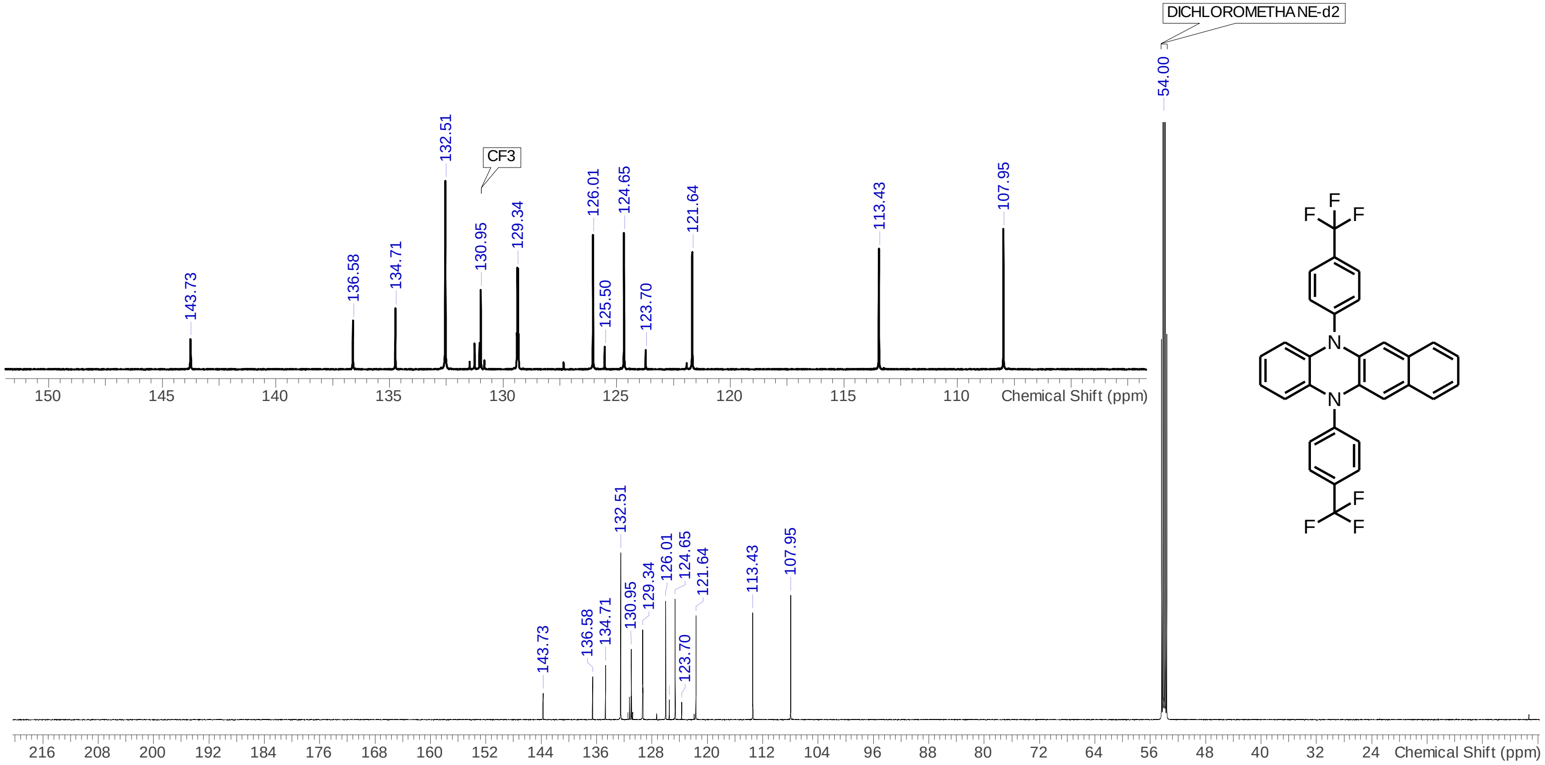

$^{1}H,^{1}H$ COSY and $^{1}H,^{1}H$ ROESY of **2**

Acquisition Time (sec)	(0.2272, 0.0284)	***Comment***	GZ 475	***Date***	07 Oct 2019 15:28:56
File Name	\\bunz22\Mitarbeiter\Gaozhan XIE\03_analytics\01-NMR\nmr-biradical01\e191007ubgz.475\3\PDATA\1\2rr				
Frequency (MHz)	(600.2438, 600.2438)			***Nucleus***	(1H, 1H)
Number of Transients	8	***Origin***	spect	***Original Points Count***	(2048, 256)
Owner	ns	***Points Count***	(2048, 2048)	***Pulse Sequence***	cosygpmfppqf
Solvent	DICHLOROMETHANE-d2			***Spectrum Type***	COSY
Sweep Width (Hz)	(9010.02, 9004.61)	***Temperature (degree C)***	22.001	***Title***	GZ 475

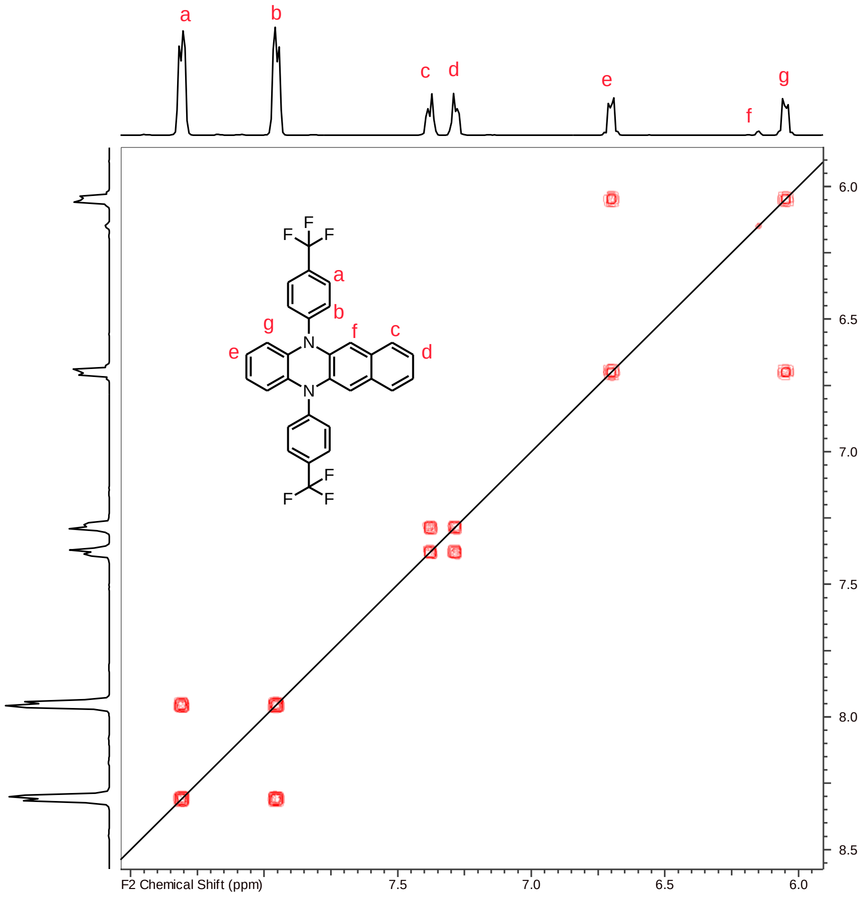

Acquisition Time (sec)	(0.1136, 0.0284)	***Comment***	GZ 475	***Date***	07 Oct 2019 18:31:50
File Name	\\bunz22\Mitarbeiter\Gaozhan XIE\03_analytics\01-NMR\nmr-biradical01\e191007ubgz.475\4\PDATA\1\2rr				
Frequency (MHz)	(600.2438, 600.2438)			***Nucleus***	(1H, 1H)
Number of Transients	16	***Origin***	spect	***Original Points Count***	(1024, 256)
Owner	ns	***Points Count***	(2048, 1024)	***Pulse Sequence***	noesygpphpp
Solvent	DICHLOROMETHANE-d2			***Spectrum Type***	NOESY
Sweep Width (Hz)	(9010.02, 9000.21)	***Temperature (degree C)***	22.001	***Title***	GZ 475

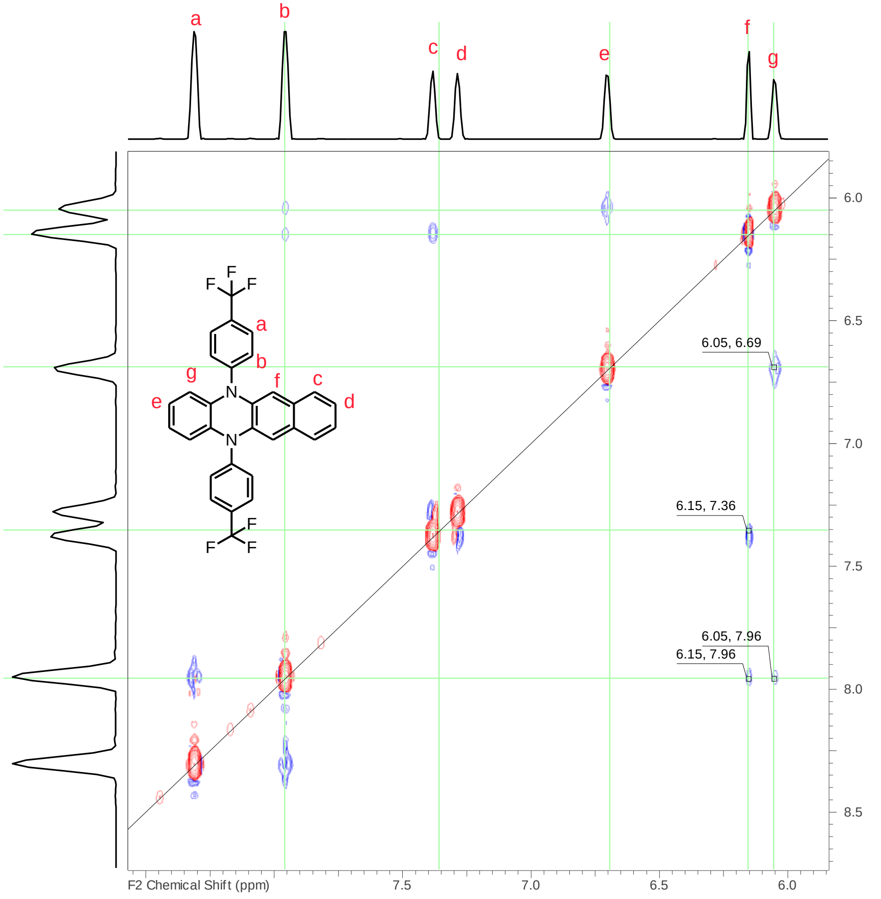

^{1}H-NMR and ^{13}C{^{1}H} NMR of **3**

Multiplets Integrals Sum	20.13	***Number of Nuclei***	30 H's

Acquisition Time (sec)	2.7263	***Comment***	GZ 446	***D***	0.5	***D1***	0.5	***DE***	10
DS	2	***Date***	10 Oct 2019 18:50:17			***Date Stamp***	10 Oct 2019 18:50:17		
File Name	\\bunz22\Mitarbeiter\Gaozhan XIE\03_analytics\01-NMR\nmr-biradical01\c191010ubgz.446\2\PDATA\1\1r							***Frequency (MHz)***	400.3300
GB	0	***INSTRUM***	<spect>	***LB***	0.3	***NS***	128	***Nucleus***	1H
Number of Transients	128	***Origin***	spect	***Original Points Count***	32768	***Owner***	ns	***PC***	1
PROBHD	<Z130030_0004 (CPP BBO 400S1 BB-H&F-D-05 LT)>			***PULPROG***	<zg30>	***Points Count***	65536	***Pulse Sequence***	zg30
Receiver Gain	812.00	***SF***	400.330049193387			***SFO1***	400.3320009	***SI***	65536
SSB	0	***SW(cyclical) (Hz)***	12019.23	***SWH***	12019.2307692308				
Solvent	DICHLOROMETHANE-d2			***Spectrum Offset (Hz)***	1985.4963	***Spectrum Type***	standard	***Sweep Width (Hz)***	12019.05
TD	65536	***TD0***	16	***TE***	295.0004	***Temperature (degree C)***	22.000	***UNC1***	<1H>
WDW	1								

User Notes	GZ 446

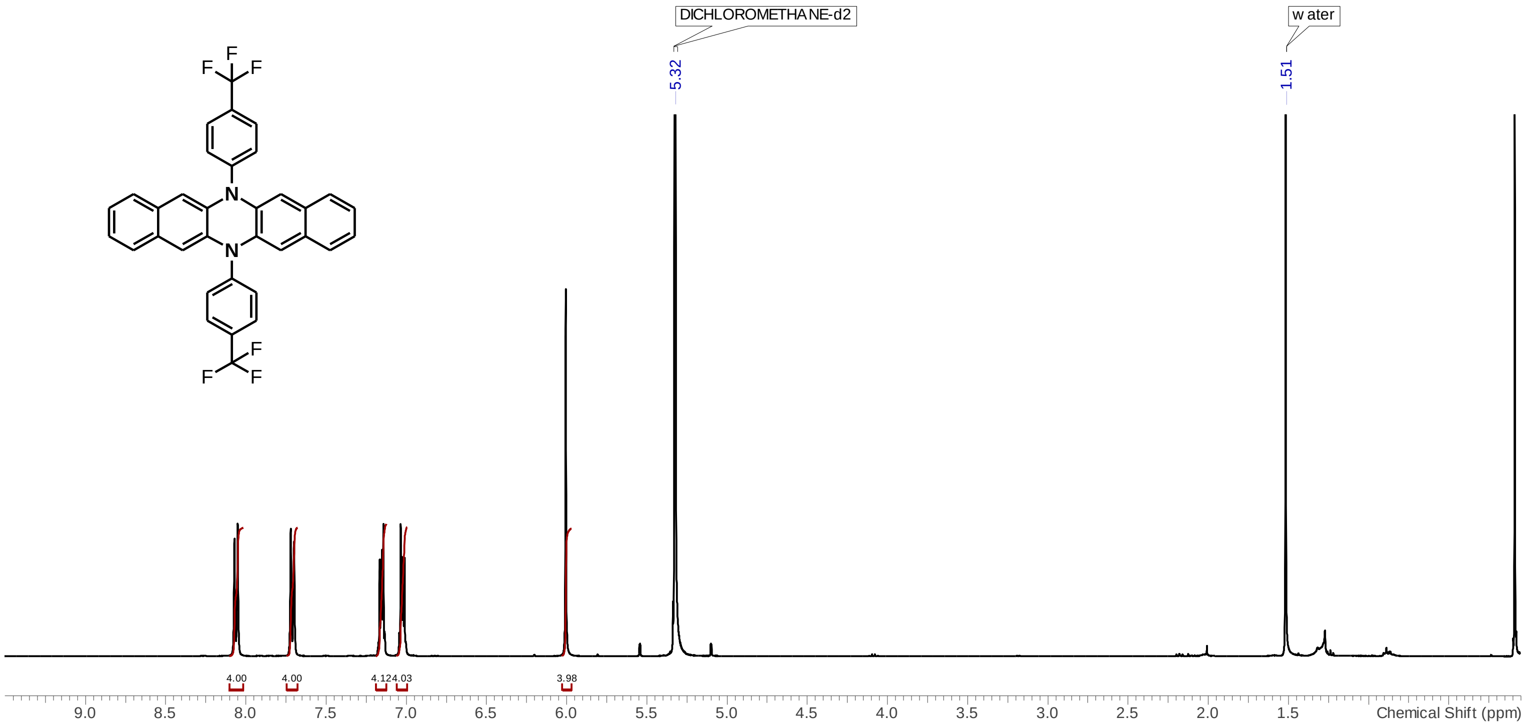

Multiplets Integrals Sum	0.00	Number of Nuclei	0 C's

Acquisition Time (sec)	1.5897	Comment	GZ 446	D	1.5	D1	1.5	DE	18
DS	2	Date	10 Oct 2019 18:42:25			Date Stamp	10 Oct 2019 18:42:25		
File Name	\\bunz22\Mitarbeiter\Gaozhan XIE\03_analytics\01-NMR\nmr-biradical01\c191010ubgz.446\1\PDATA\1\1r							Frequency (MHz)	100.6631
GB	0	INSTRUM	<spect>	LB	1	NS	4096	Nucleus	13C
Number of Transients	4096	Origin	spect	Original Points Count	49066	Owner	ns	PC	1.4
PROBHD	<Z130030_0004 (CPP BBO 400S1 BB-H&F-D-05 LT)>			PULPROG	<zgpg30>	Points Count	65536	Pulse Sequence	zgpg30
Receiver Gain	2050.00	SF	100.663059	SFO1	100.67413193649			SI	65536
SSB	0	SW(cyclical) (Hz)	30864.20	SWH	30864.1975308642				
Solvent	DICHLOROMETHANE-d2			Spectrum Offset (Hz)	11135.0059	Spectrum Type	standard	Sweep Width (Hz)	30863.73
TD	98132	TD0	512	TE	295.0007	Temperature (degree C)	22.001	UNC1	<13C>
WDW	1								

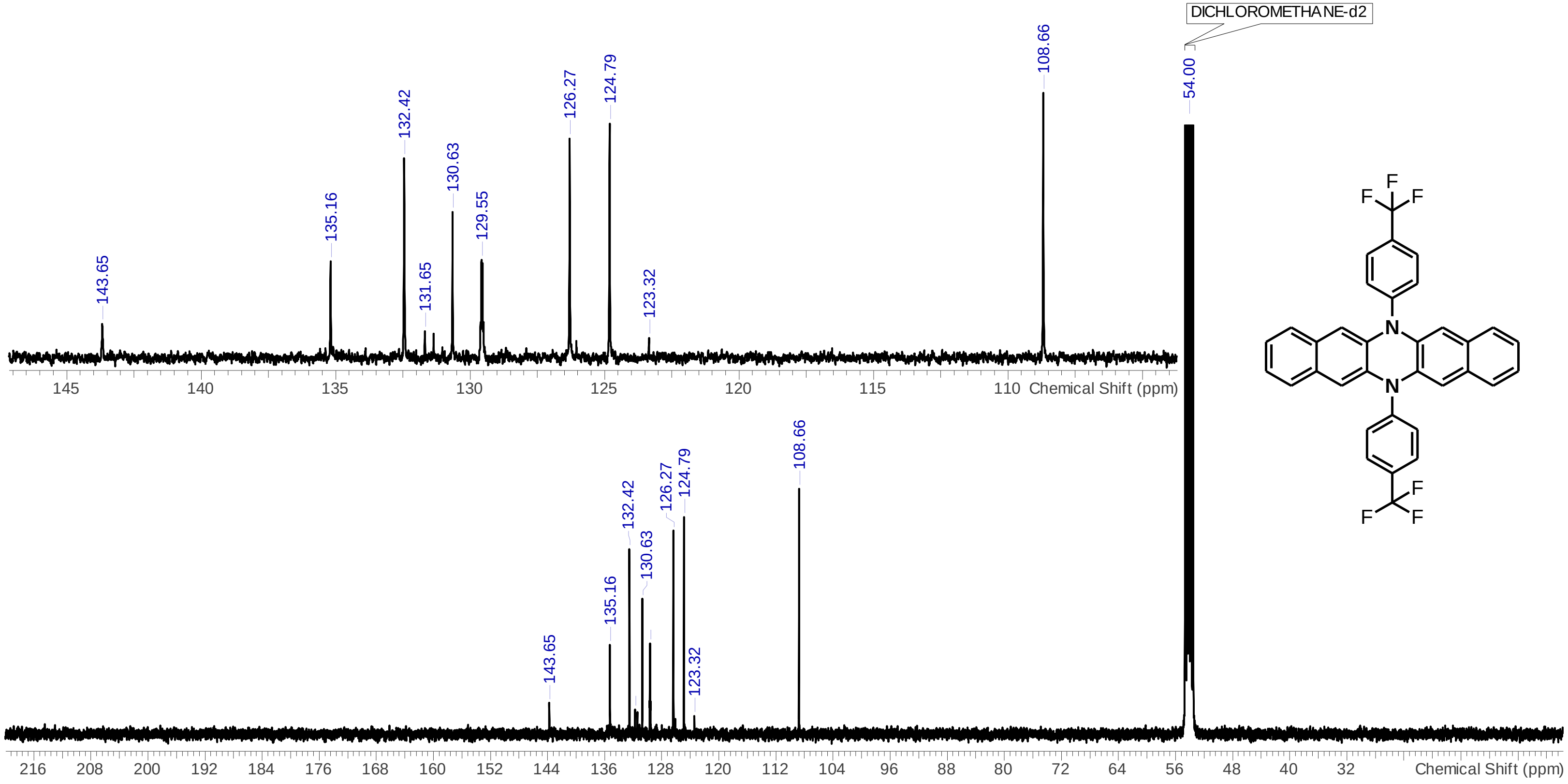

$^{1}H,^{1}H$ COSY and $^{1}H,^{1}H$ ROESY of **3**

Acquisition Time (sec)	(0.3408, 0.0426)	***Comment***	GZ 446	***Date***	10 Oct 2019 19:14:22
File Name	\\bunz22\Mitarbeiter\Gaozhan XIE\03_analytics\01-NMR\nmr-biradical01\c191010ubgz.446\3\PDATA\1\2rr				
Frequency (MHz)	(400.3300, 400.3300)			***Nucleus***	(1H, 1H)
Number of Transients	8	***Origin***	spect	***Original Points Count***	(2048, 256)
Owner	ns	***Points Count***	(2048, 2048)	***Pulse Sequence***	cosygpmfppqf
Solvent	DICHLOROMETHANE-d2			***Spectrum Type***	COSY
Sweep Width (Hz)	(6006.68, 6006.68)	***Temperature (degree C)***	22.000	***Title***	GZ 446

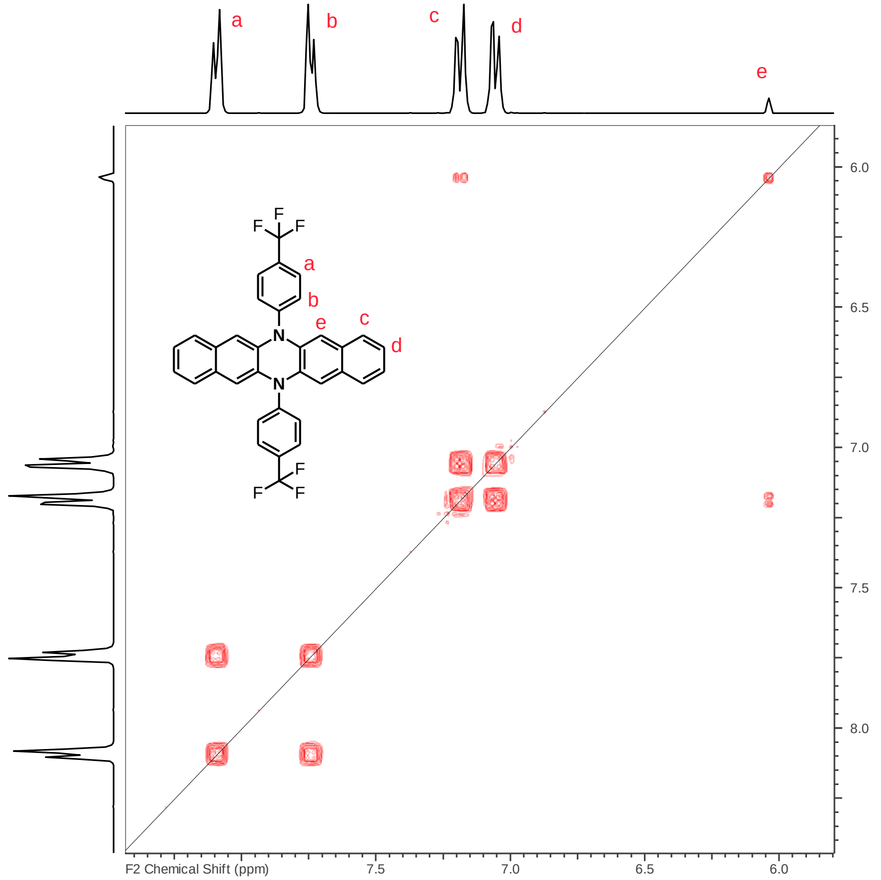

Acquisition Time (sec)	(0.3408, 0.0426)	***Comment***	GZ 446	***Date***	11 Oct 2019 08:01:30
File Name	\\bunz22\Mitarbeiter\Gaozhan XIE\03_analytics\01-NMR\nmr-biradical01\c191010ubgz.446\4\PDATA\1\2rr				
Frequency (MHz)	(400.3300, 400.3300)			***Nucleus***	(1H, 1H)
Number of Transients	16	***Origin***	spect	***Original Points Count***	(2048, 256)
Owner	ns	***Points Count***	(2048, 2048)	***Pulse Sequence***	roesyetgp.2
Solvent	DICHLOROMETHANE-d2			***Spectrum Type***	ROESY
Sweep Width (Hz)	(6006.68, 6006.68)	***Temperature (degree C)***	22.000	***Title***	GZ 446

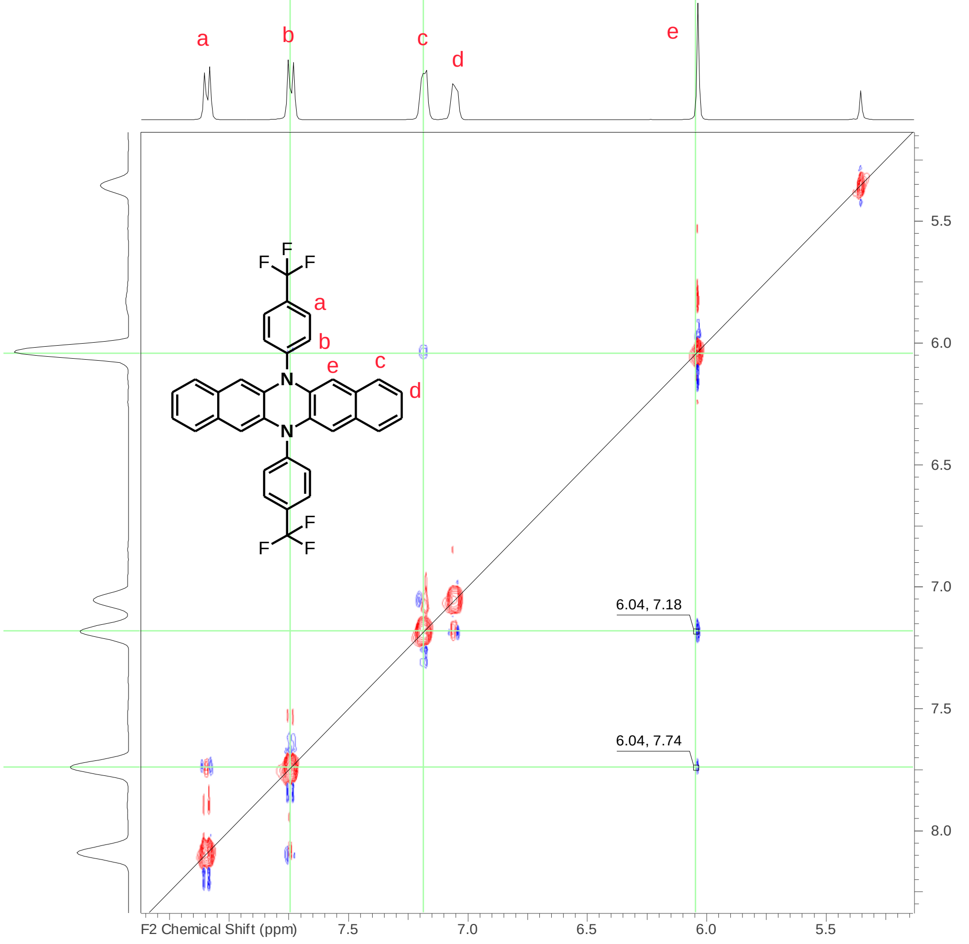

S8. Mass Spectra

$1^{+\bullet}$

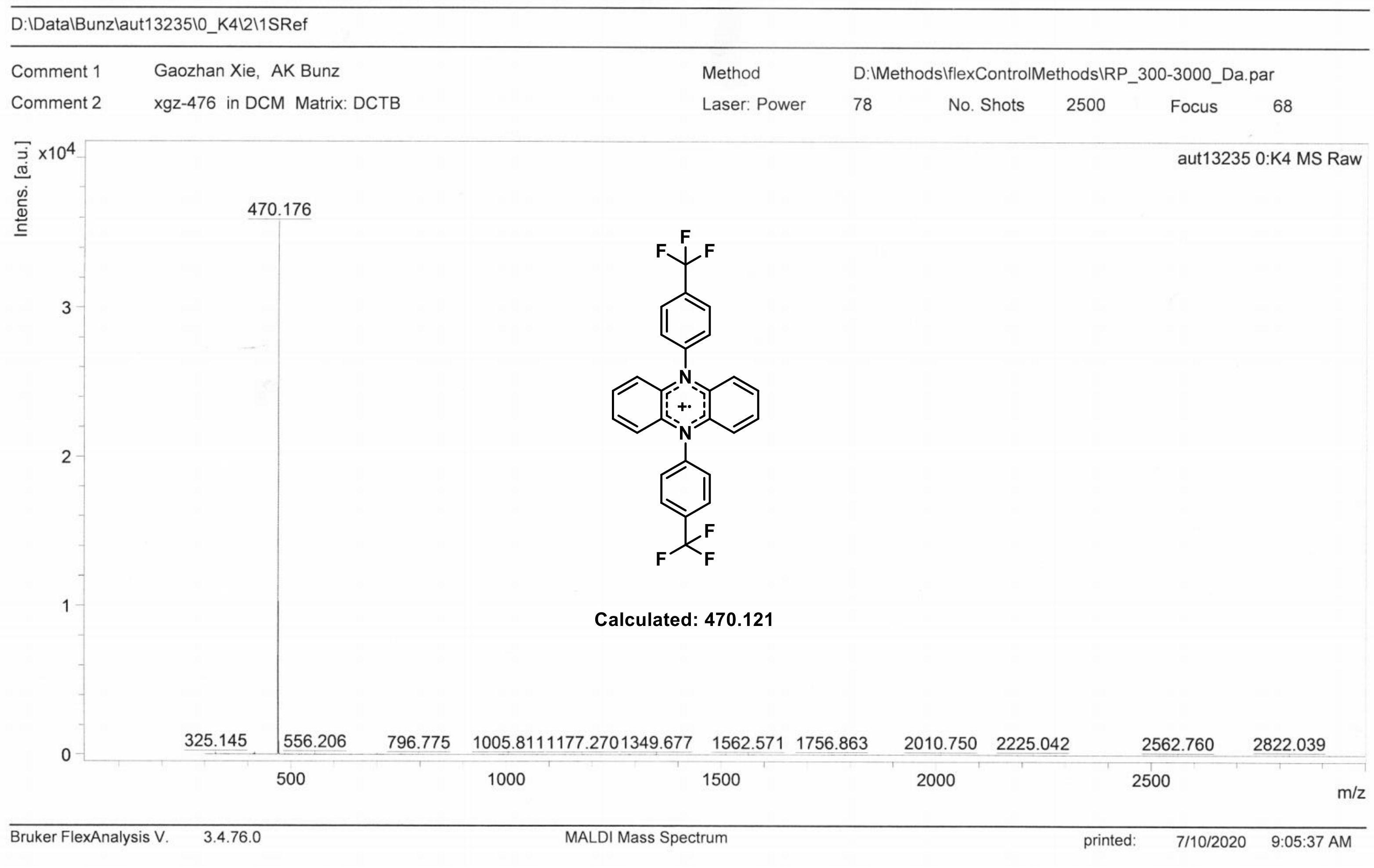

2

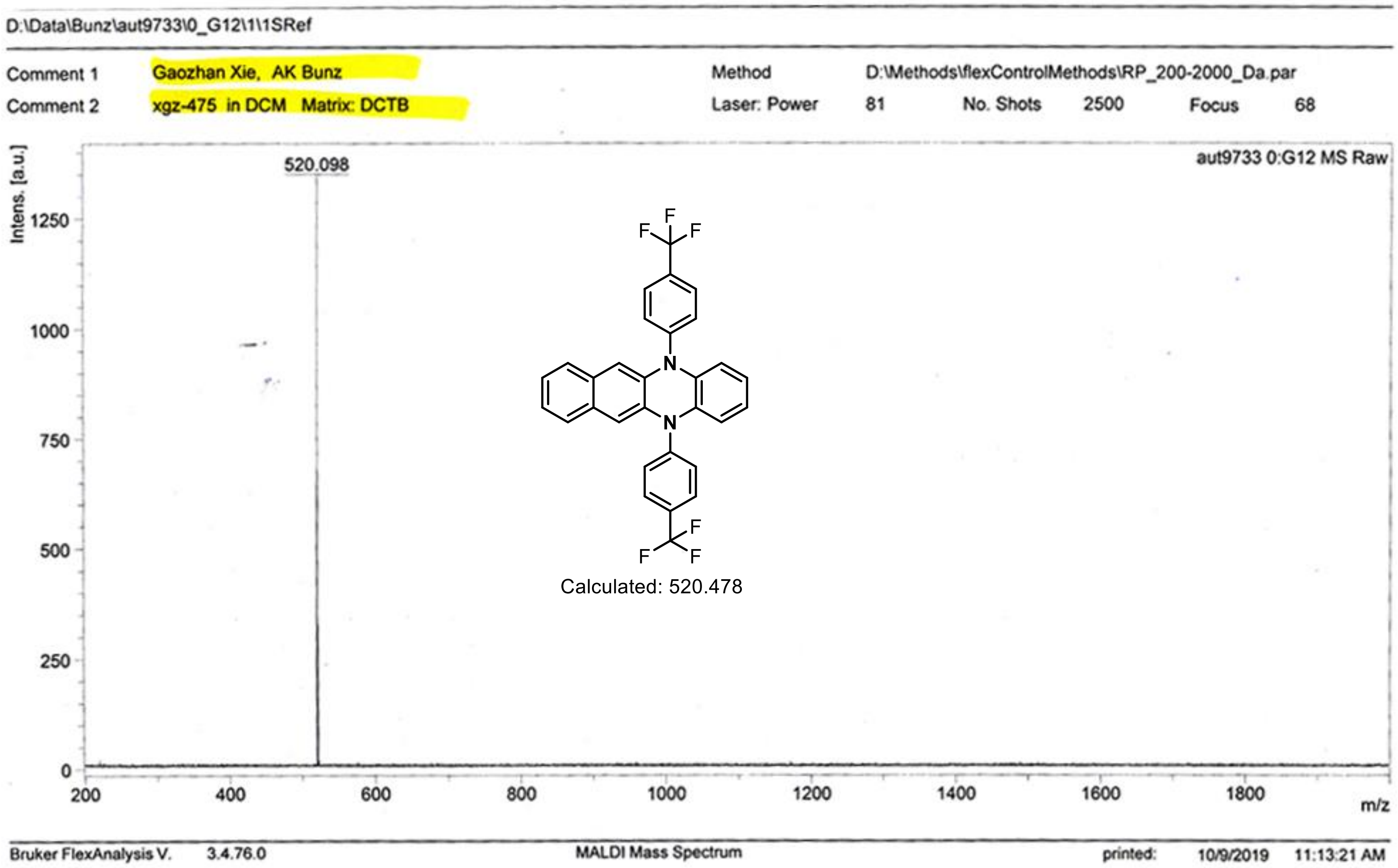
D:\Data\Bunz\aut9733\0_G12\1\1SRef
Comment 1 Gaozhan Xie, AK Bunz
Comment 2 xgz-475 in DCM Matrix: DCTB
Method D:\Methods\flexControlMethods\RP_200-2000_Da.par
Laser: Power 81 No. Shots 2500 Focus 68
Intens. [a.u.]
520.098
aut9733 0:G12 MS Raw
1250
1000
750
500
250
0
200
400
600
800
1000
1200
1400
1600
1800
m/z
F
F
F
N
N
Calculated: 520.478
Bruker FlexAnalysis V. 3.4.76.0
MALDI Mass Spectrum
printed: 10/9/2019 11:13:21 AM

$2^{+\bullet}$

D:\Data\Bunz\aut13234\0_K3\2\1SRef

Comment 1	Gaozhan Xie, AK Bunz	Method	D:\Methods\flexControlMethods\RP_300-3000_Da.par				
Comment 2	xgz-486 in DCM Matrix: DCTB	Laser: Power	78	No. Shots	2500	Focus	68

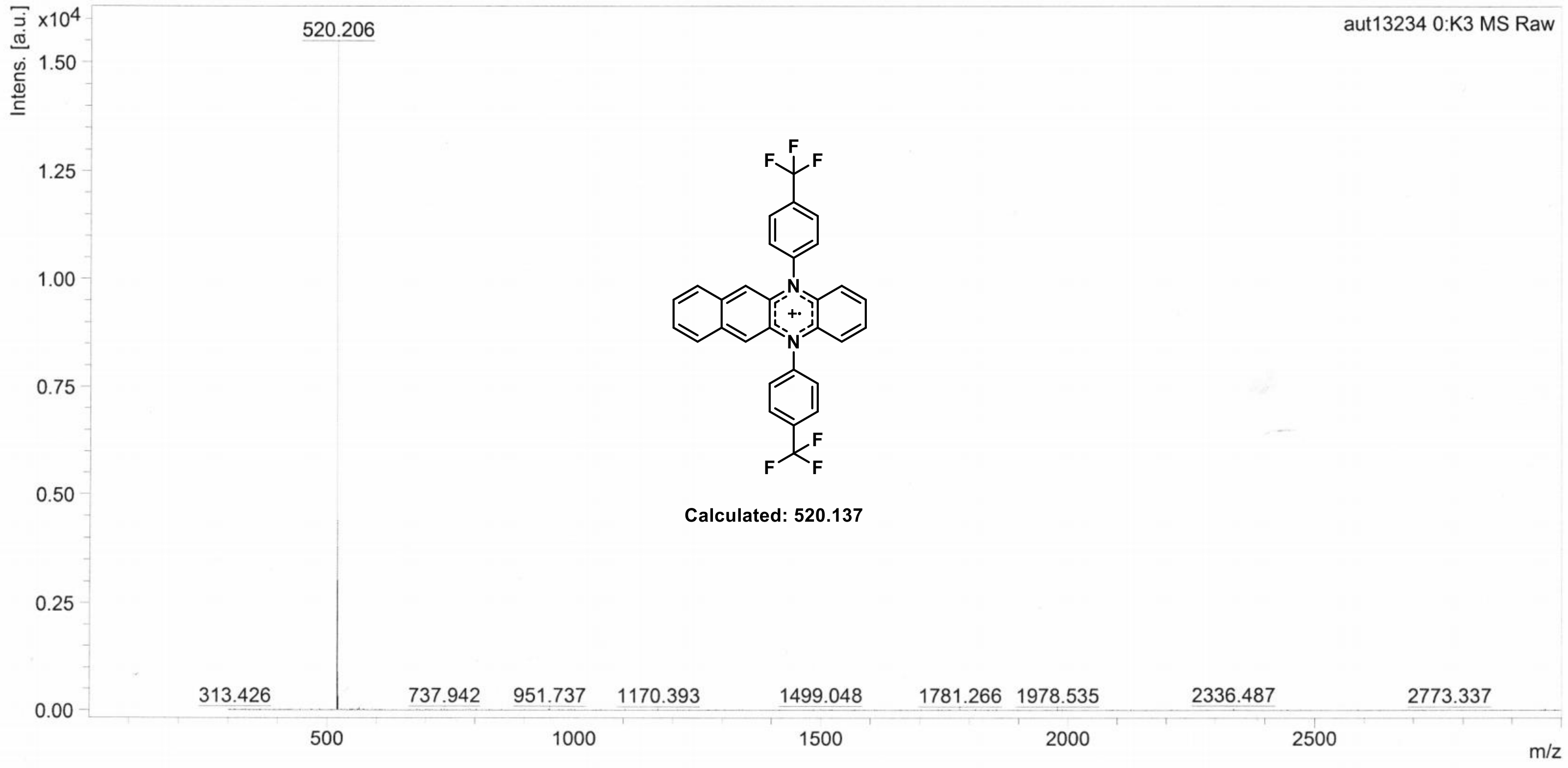

Bruker FlexAnalysis V. 3.4.76.0 MALDI Mass Spectrum printed: 7/10/2020 9:06:59 AM

3

D:\Data\Bunz\aut9408\0_L9\1\1SRef

Comment 1	Gaozhan Xie, AK Bunz	Method	D:\Methods\flexControlMethods\RP_300-3000_Da.par
Comment 2	xgz-446 in DCM Matrix: DCTB	Laser: Power	72 No. Shots 2500 Focus 68

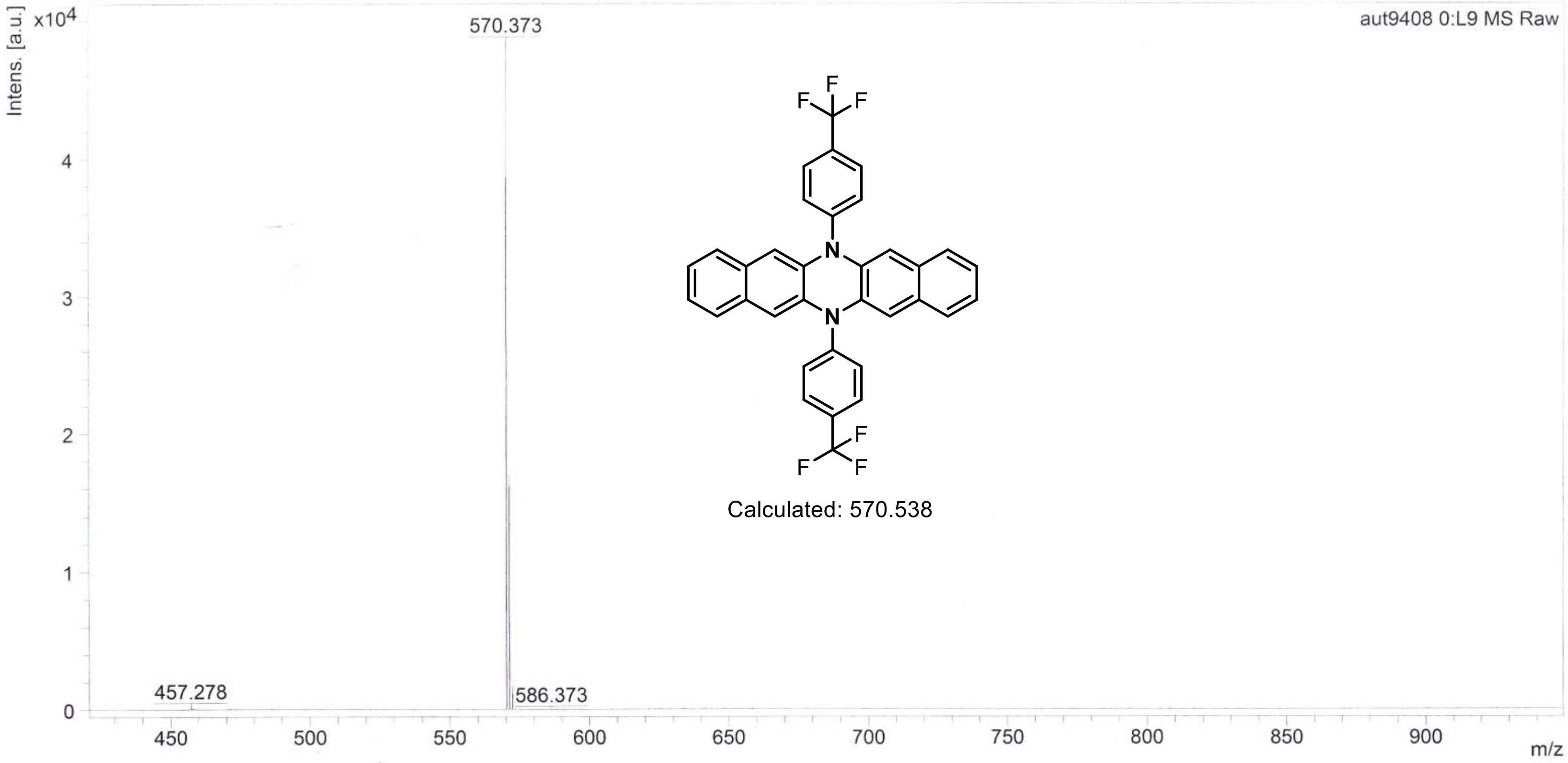

Bruker FlexAnalysis V. 3.4.76.0 MALDI Mass Spectrum printed: 9/17/2019 1:29:49 PM

$3^{+\bullet}$

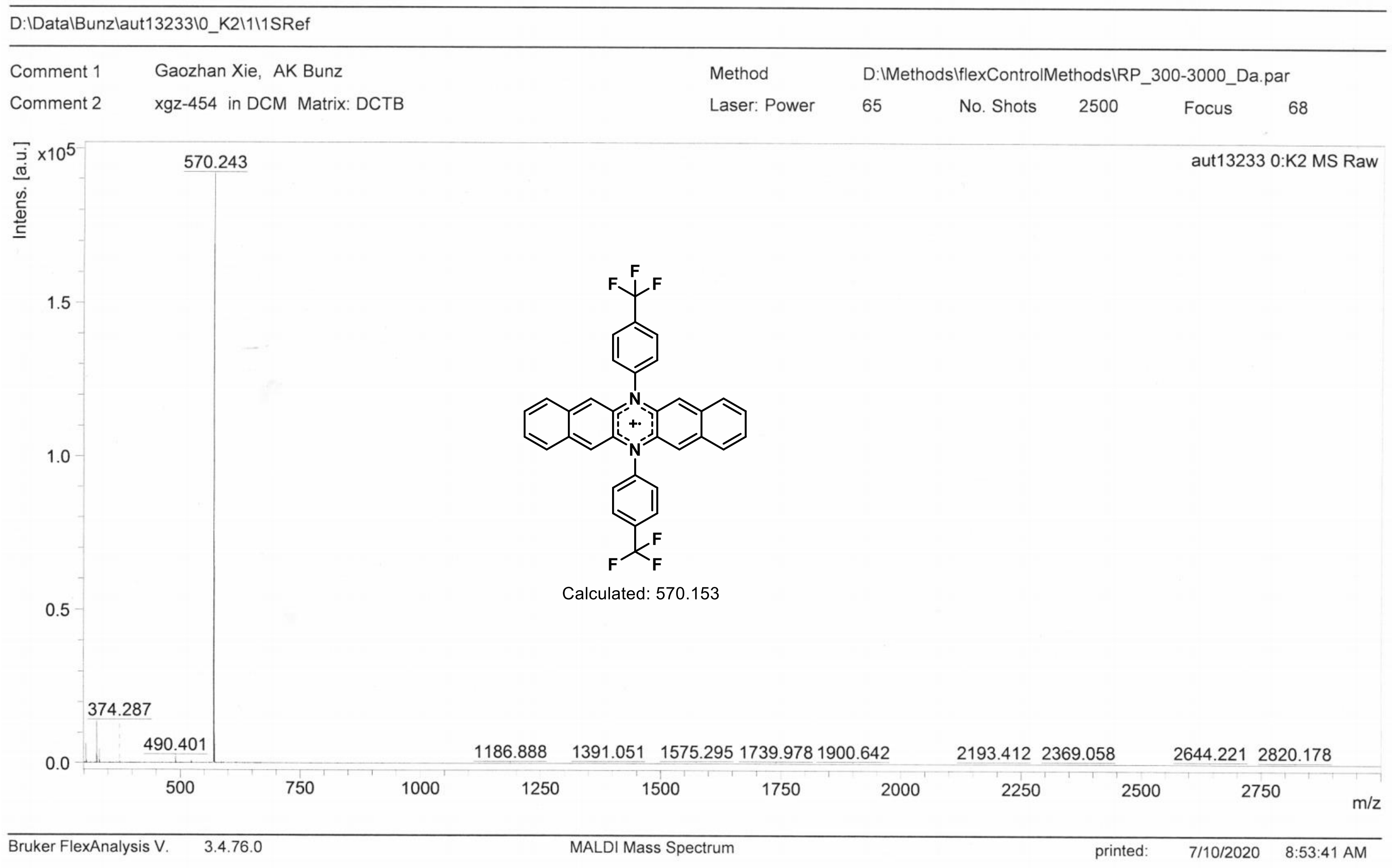
D:\Data\Bunz\aut13233\0_K2\1\1SRef
Comment 1
Gaozhan Xie, AK Bunz
Comment 2
xgz-454 in DCM Matrix: DCTB
Method
D:\Methods\flexControlMethods\RP_300-3000_Da.par
Laser: Power
65
No. Shots
2500
Focus
68
Intens. [a.u.]
x10^5
aut13233 0:K2 MS Raw
570.243
374.287
490.401
1186.888
1391.051
1575.295
1739.978
1900.642
2193.412
2369.058
2644.221
2820.178
1.5
1.0
0.5
0.0
500
750
1000
1250
1500
1750
2000
2250
2500
2750
m/z
F
F
F
N
N
+·
F
F
F
Calculated: 570.153
Bruker FlexAnalysis V.
3.4.76.0
MALDI Mass Spectrum
printed:
7/10/2020
8:53:41 AM

S9. Infrared Spectra

$1^{+\bullet}$

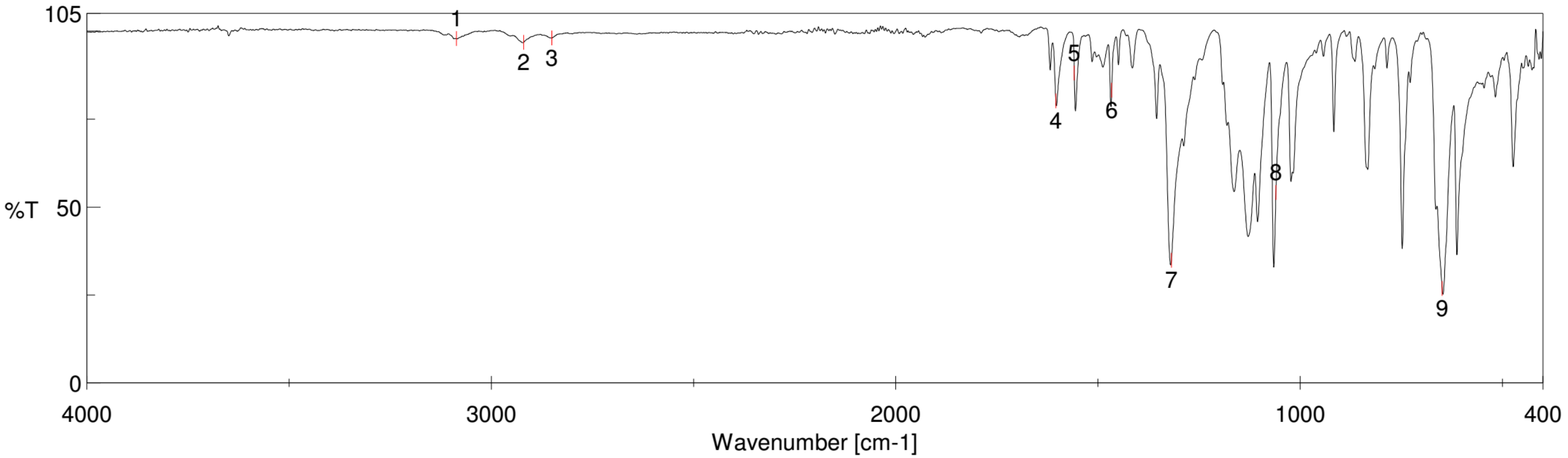

[Result of Peak Picking]

No.	Position	Intensity	No.	Position	Intensity	No.	Position	Intensity
1	3086.03	97.8123	2	2920.18	96.7457	3	2850.76	97.9991
4	1604.48	80.1395	5	1558.68	88.0431	6	1466.12	83.1669
7	1318.59	34.8232	8	1060.17	54.1252	9	649.411	26.8046

2

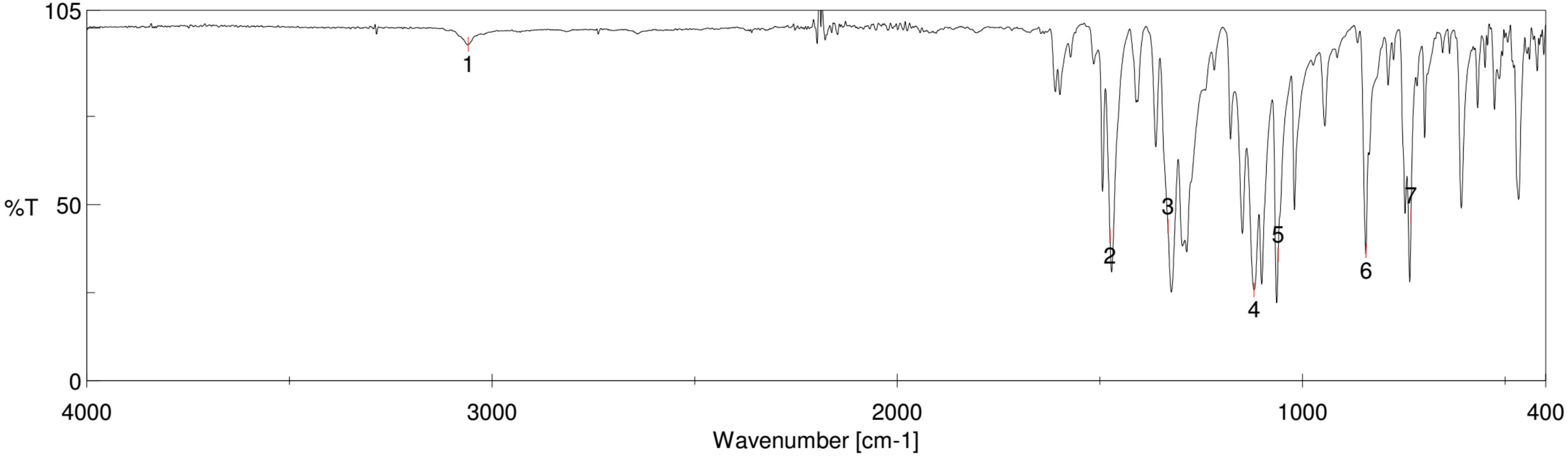

[Result of Peak Picking]

No.	Position	Intensity	No.	Position	Intensity	No.	Position	Intensity
1	3058.55	95.327	2	1475.28	41.0297	3	1332.09	43.73
4	1119.96	25.7427	5	1060.17	35.6636	6	843.222	36.8177
7	732.335	46.6044						

$2^{+\bullet}$

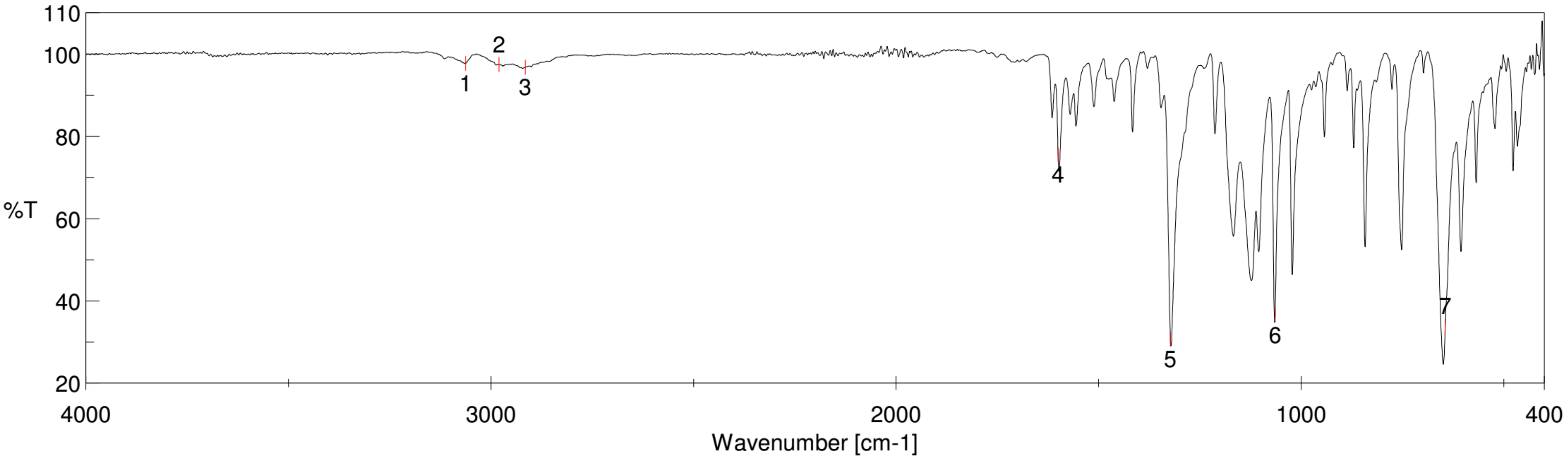

[Result of Peak Picking]

No.	Position	Intensity	No.	Position	Intensity	No.	Position	Intensity
1	3062.89	97.6591	2	2979.96	97.4065	3	2915.36	96.7177
4	1600.15	75.4209	5	1322.93	30.6598	6	1064.51	36.4919
7	644.59	33.8453						

3

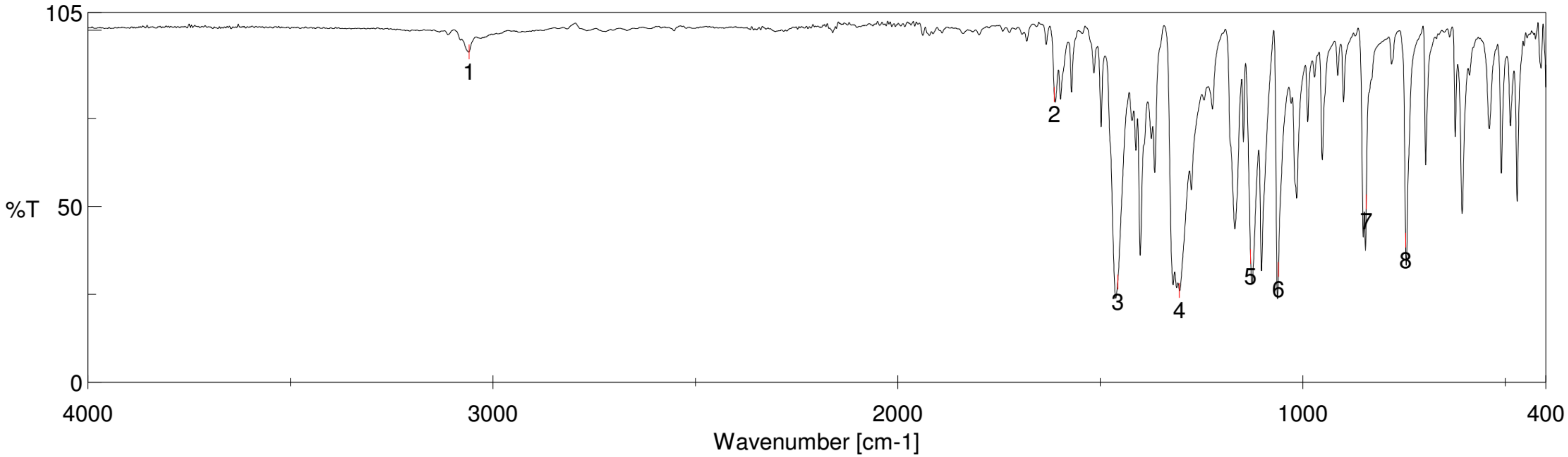

[Result of Peak Picking]

No.	Position	Intensity	No.	Position	Intensity	No.	Position	Intensity
1	3058.55	93.7856	2	1613.64	81.6765	3	1456.96	28.4393
4	1304.61	26.0339	5	1129.12	35.7053	6	1060.17	31.9819
7	843.222	51.1824	8	746.317	40.375			

$3^{+\bullet}$

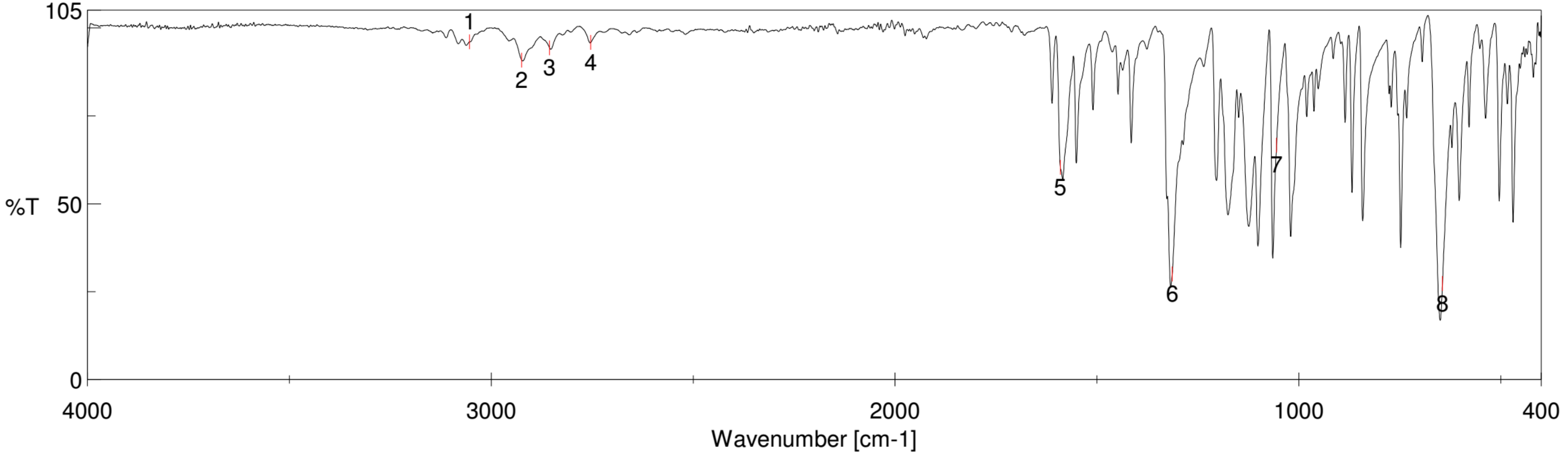

[Result of Peak Picking]

No.	Position	Intensity	No.	Position	Intensity	No.	Position	Intensity
1	3053.73	95.9853	2	2924.52	90.7606	3	2855.58	94.3088
4	2753.85	95.7972	5	1590.99	60.347	6	1313.77	29.9645
7	1055.35	66.6398	8	644.59	27.2492			

S10. Crystals Structures

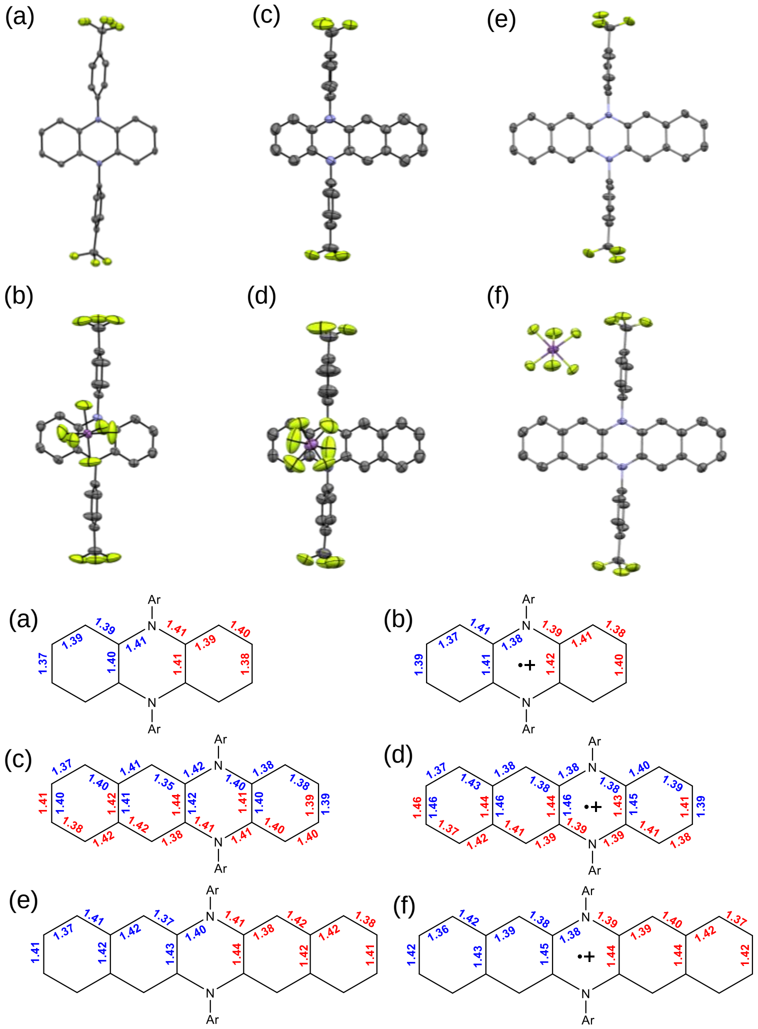

Figure S8. Single crystal structures and averaged bond lengths (bottom, average of all equivalent bonds, blue: derived from the crystal structures; red: calculated at the DFT/B3LYP/6-311+G** level of theory) of a) **1**; b) $\mathbf{1}^{+\cdot}$; c) **2**; d) $\mathbf{2}^{+\cdot}$; e) **3**; f) $\mathbf{3}^{+\cdot}$. The single crystal data of **1** was originally reported in ref. 5.

$1^{+\bullet}$

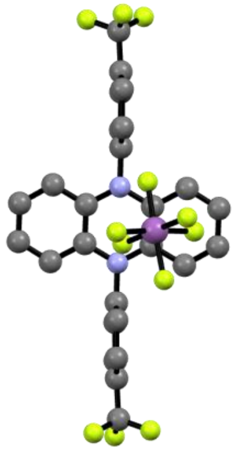

Identification code	gxi24
Empirical formula	$C_{27}H_{18}Cl_2F_{12}N_2Sb$
Formula weight	791.08
Temperature	200(2) K
Wavelength	0.71073 Å
Crystal system	monoclinic
Space group	C2/c
Z	4
Unit cell dimensions	a = 15.7925(10) Å α = 90 deg.
	b = 23.3446(15) Å β = 92 deg.
	c =8.0504(5) Å γ = 90 deg.
Volume	2963.9(3) $Å^3$
Density (calculated)	1.77 g/cm^3
Absorption coefficient	1.21 mm^{-1}
Crystal shape	plate
Crystal size	0.132 x 0.111 x 0.022 mm^3
Crystal colour	green
Theta range for data collection	1.6 to 30.5 deg.
Index ranges	$-22\leq h\leq 22$, $-31\leq k\leq 33$, $-11\leq l\leq 11$
Reflections collected	18810
Independent reflections	4530 (R(int) = 0.0270)
Observed reflections	3811 ($I > 2\sigma(I)$)
Absorption correction	Semi-empirical from equivalents
Max. and min. transmission	0.96 and 0.89
Refinement method	Full-matrix least-squares on F^2
Data/restraints/parameters	4530 / 8 / 213
Goodness-of-fit on F^2	1.06
Final R indices (I>2sigma(I))	R1 = 0.038, wR2 = 0.094
Largest diff. peak and hole	0.57 and -0.51 $eÅ^{-3}$

2

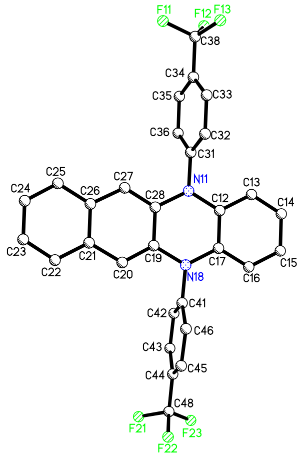

Identification code	gxi20	
Empirical formula	$C_{30}H_{18}F_6N_2$	
Formula weight	520.46	
Temperature	200(2) K	
Wavelength	1.54178 Å	
Crystal system	monoclinic	
Space group	$P2_1$	
Z	4	
Unit cell dimensions	a = 13.0758(11) Å	α = 90 deg.
	b = 9.4675(6) Å	β = 100 deg.
	c = 19.5081(13) Å	γ = 90 deg.
Volume	2377.3(3) $Å^3$	
Density (calculated)	1.45 g/cm^3	
Absorption coefficient	1.02 mm^{-1}	
Crystal shape	?	
Crystal size	0.097 x 0.068 x 0.042 mm^3	
Crystal colour	pale yellow	
Theta range for data collection	3.4 to 57.9 deg.	
Index ranges	$-14 \le h \le 14$, $-10 \le k \le 10$, $-12 \le l \le 21$	
Reflections collected	16536	
Independent reflections	6204 (R(int) = 0.0565)	
Observed reflections	3445 ($I > 2\sigma(I)$)	
Absorption correction	Semi-empirical from equivalents	
Max. and min. transmission	1.42 and 0.71	
Refinement method	Full-matrix least-squares on F^2	
Data/restraints/parameters	6204 / 835 / 711	
Goodness-of-fit on F^2	0.99	
Final R indices (I>2sigma(I))	R1 = 0.096, wR2 = 0.231	
Absolute structure parameter	0.2(2)	
Largest diff. peak and hole	0.58 and -0.37 $eÅ^{-3}$	

$2^{+\bullet}$

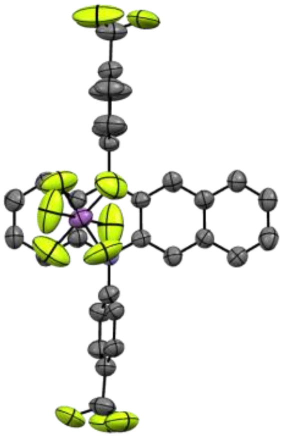

Identification code	gxi22	
Empirical formula	$C_{31.50}H_{21}F_{12}N_2O_{0.50}Sb$	
Formula weight	785.25	
Temperature	200(2) K	
Wavelength	1.54178 Å	
Crystal system	orthorhombic	
Space group	Aba2	
Z	8	
Unit cell dimensions	a = 46.518(4) Å	α = 90 deg.
	b = 16.4075(15) Å	β = 90 deg.
	c = 8.0281(6) Å	γ = 90 deg.
Volume	6127.4(9) $Å^3$	
Density (calculated)	1.70 g/cm^3	
Absorption coefficient	8.07 mm^{-1}	
Crystal shape	plate	
Crystal size	0.125 x 0.071 x 0.020 mm^3	
Crystal colour	orange	
Theta range for data collection	5.7 to 63.7 deg.	
Index ranges	$-52 \le h \le 54$, $-18 \le k \le 8$, $-8 \le l \le 9$	
Reflections collected	9129	
Independent reflections	4182 (R(int) = 0.0895)	
Observed reflections	2513 ($I > 2\sigma(I)$)	
Absorption correction	Semi-empirical from equivalents	
Max. and min. transmission	3.60 and 0.62	
Refinement method	Full-matrix least-squares on F^2	
Data/restraints/parameters	4182 / 1062 / 481	
Goodness-of-fit on F^2	0.97	
Final R indices (I>2sigma(I))	R1 = 0.070, wR2 = 0.170	
Absolute structure parameter	0.00(2)	
Largest diff. peak and hole	0.49 and -0.87 $eÅ^{-3}$	

3

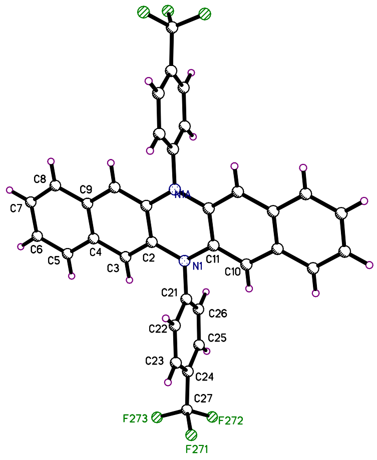

Identification code	gxi17
Empirical formula	$C_{34}H_{20}F_6N_2$
Formula weight	570.52
Temperature	200(2) K
Wavelength	0.71073 Å
Crystal system	Monoclinic
Space group	$P2_1/c$
Z	2
Unit cell dimensions	a = 13.0273(7) Å α = 90 deg.
	b =9.5645(5) Å β =96.985 deg.
	c = 10.6532(6) Å γ = 90 deg.
Volume	1317.53(12) $Å^3$
Density (calculated)	1.44 g/cm^3
Absorption coefficient	0.11 mm^{-1}
Crystal shape	brick
Crystal size	0.132 x 0.072 x 0.053 mm^3
Crystal colour	yellow
Theta range for data collection	1.6 to 31.5 deg.
Index ranges	$-19 \le h \le 18$, $-14 \le k \le 13$, $-15 \le l \le 15$
Reflections collected	18491
Independent reflections	4357 (R(int) = 0.0567)
Observed reflections	2592 (I > 2\s(I))
Absorption correction	Semi-empirical from equivalents
Refinement method	Full-matrix least-squares on F^2
Data/restraints/parameters	4357 / 0 / 190
Goodness-of-fit on F^2	1.03
Final R indices ($I>2\sigma(I)$)	R1 = 0.061, wR2 = 0.148
Largest diff. peak and hole	0.44 and -0.41 $eÅ^{-3}$

$3^{+\bullet}$

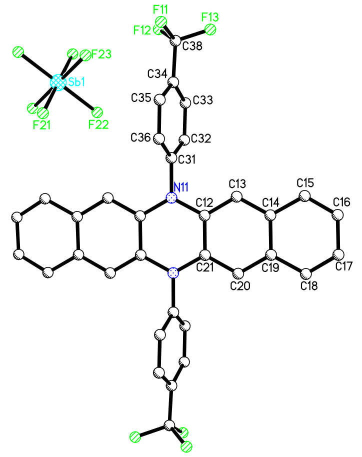

Identification code	gxi18	
Empirical formula	C34H20F12N2Sb	
Formula weight	806.27	
Temperature	200(2) K	
Wavelength	1.54178 Å	
Crystal system	triclinic	
Space group	$P\bar{1}$	
Z	1	
Unit cell dimensions	a = 8.4529(8) Å	□□ = 97.542(8) deg.
	b = 8.4694(8) Å	□□ = 91.735(8) deg.
	c = 11.8279(13) Å	□□ = 113.939(7) deg.
Volume	763.97(14) Å3	
Density (calculated)	1.75 g/cm3	
Absorption coefficient	8.10 mm-1	
Crystal shape	plate	
Crystal size	0.077 x 0.060 x 0.012 mm3	
Crystal colour	violet	
Theta range for data collection	3.8 to 67.0 deg.	
Index ranges	-10□h□5, -9□k□10, -14□l□13	
Reflections collected	6402	
Independent reflections	2603 (R(int) = 0.0397)	
Observed reflections	2086 (I > 2□(I))	
Absorption correction	Semi-empirical from equivalents	
Max. and min. transmission	1.41 and 0.67	
Refinement method	Full-matrix least-squares on F2	
Data/restraints/parameters	2603 / 79 / 251	
Goodness-of-fit on F2	1.15	
Final R indices (I>2sigma(I))	R1 = 0.048, wR2 = 0.094	
Largest diff. peak and hole	0.74 and -0.86 eÅ-3	

S11. Cartesian Coordinates of Computational Molecules

Coordinates of **1**:

0 1

C	0.72104900	-3.65327300	-0.24054300
C	-0.66422100	-3.66410800	-0.23954900
C	-1.36637400	-2.45583200	-0.16925500
C	-0.69793400	-1.23368100	-0.09362600
C	0.71701000	-1.22264600	-0.09411200
C	1.40434700	-2.43419200	-0.17100400
N	-1.39600100	-0.01090700	0.00731400
C	-0.71701400	1.22264500	-0.09381200
C	0.69792900	1.23368000	-0.09340800
N	1.39600200	0.01088400	0.00723300
C	-1.40435600	2.43420700	-0.17041400
C	-0.72106200	3.65330300	-0.23974100
C	0.66420800	3.66413600	-0.23882700
C	1.36636500	2.45584600	-0.16882600
C	2.82950100	0.02150100	0.03126200
C	-2.82949800	-0.02152400	0.03140700
C	3.49815300	0.02412300	1.25512600
C	4.88969000	0.03360700	1.29018000
C	5.60998700	0.04391300	0.09535900
C	4.94492600	0.04070100	-1.13240500
C	3.55440000	0.03069800	-1.16202900
C	-3.55445200	-0.03051200	-1.16185400
C	-4.94497600	-0.04051000	-1.13216800
C	-5.60998200	-0.04392200	0.09562600
C	-4.88963100	-0.03382700	1.29041600
C	-3.49809500	-0.02435200	1.25530000
C	7.11207800	0.00152000	0.12576900
F	7.62463900	0.54913100	1.25137800
F	7.58636000	-1.27371900	0.07207100
F	7.66918400	0.65546500	-0.92033200
C	-7.11207200	-0.00151700	0.12610000
F	-7.66922400	-0.65541500	-0.92000800
F	-7.62459500	-0.54916700	1.25170500
F	-7.58634300	1.27372900	0.07246700
H	1.28454300	-4.57719000	-0.29353200
H	-1.21324600	-4.59674700	-0.29161100
H	-2.44765900	-2.47071300	-0.16559800
H	2.48573100	-2.43220100	-0.16865400
H	-2.48574000	2.43221500	-0.16800100
H	-1.28455900	4.57723000	-0.29250400
H	1.21323000	4.59678600	-0.29073100
H	2.44765000	2.47072600	-0.16523400
H	2.92661500	0.02005700	2.17511300
H	5.40664600	0.03858400	2.24117800
H	5.50550600	0.05154000	-2.05854800
H	3.02812600	0.03173600	-2.10881100
H	-3.02822100	-0.03138900	-2.10865900
H	-5.50559700	-0.05118700	-2.05828800
H	-5.40654500	-0.03896100	2.24143500
H	-2.92651600	-0.02044800	2.17526200

Coordinates of $\mathbf{1}^{+\bullet}$:

1 2

C	0.72498300	-3.62489100	-0.00046600
C	-0.67823900	-3.63394000	-0.00051000
C	-1.38315000	-2.44657900	-0.00052900
C	-0.70341700	-1.21553200	-0.00051200
C	0.71913800	-1.20634700	-0.00047700
C	1.41460900	-2.42859300	-0.00044800
N	-1.39185300	-0.00900600	-0.00046800
C	-0.71913800	1.20633500	-0.00052900
C	0.70341600	1.21552000	-0.00048600
N	1.39185300	0.00899400	-0.00040500
C	-1.41460900	2.42858100	-0.00064000
C	-0.72498300	3.62487900	-0.00071300
C	0.67823900	3.63392700	-0.00066600
C	1.38315000	2.44656700	-0.00055000
C	2.84163700	0.01795800	-0.00016800
C	-2.84163700	-0.01796900	-0.00017000
C	3.52161200	0.02329600	1.21429100
C	4.91296600	0.03304800	1.21280800
C	5.60305000	0.03989900	0.00033200
C	4.91340000	0.03280900	-1.21239600
C	3.52204900	0.02305100	-1.21438700
C	-3.52212500	-0.02339700	-1.21436100
C	-4.91346300	-0.03315700	-1.21227800
C	-5.60305100	-0.03989400	0.00051100
C	-4.91290200	-0.03270200	1.21292600
C	-3.52153500	-0.02296800	1.21431600
C	7.10921300	0.00138000	0.00057900
F	7.63711200	0.60482500	1.08808700
F	7.57755100	-1.27371900	0.00026100
F	7.63746800	0.60548600	-1.08639900
C	-7.10921300	-0.00136100	0.00064300
F	-7.63740200	-0.60860600	-1.08464900
F	-7.63719000	-0.60165000	1.08982700
F	-7.57753900	1.27373600	-0.00344400
H	1.27486100	-4.55739300	-0.00043900
H	-1.21600900	-4.57347500	-0.00051900
H	-2.46312700	-2.45869700	-0.00054700
H	2.49466800	-2.42694800	-0.00040500
H	-2.49466800	2.42693600	-0.00068300
H	-1.27486100	4.55738100	-0.00081700
H	1.21600900	4.57346300	-0.00073200
H	2.46312700	2.45868500	-0.00052500
H	2.97119200	0.02165300	2.14670000
H	5.45174200	0.04100700	2.15121300
H	5.45251800	0.04058400	-2.15060700
H	2.97196900	0.02121100	-2.14699800
H	-2.97209600	-0.02183500	-2.14700300
H	-5.45264200	-0.04122700	-2.15045800
H	-5.45161900	-0.04036100	2.15136100
H	-2.97106500	-0.02105400	2.14669600

Coordinates of **2**:

0 1

C	-0.65760600	-4.21522100	-0.01640800
C	0.72961600	-4.20336600	-0.01696300
C	-1.36010600	-3.00784000	-0.01238200
C	-0.69138200	-1.78166200	-0.00878700
C	0.72193300	-1.76958200	-0.00912200
C	1.41145000	-2.98419300	-0.01336300
N	-1.39200400	-0.55888400	-0.00417300
C	-0.72518300	0.67789400	-0.00521000
C	0.71363300	0.69021500	-0.00520500
N	1.40152800	-0.53491900	-0.00455300
C	-1.40886200	1.87396800	-0.00553100
C	-0.73912600	3.12830400	-0.00548300
C	0.68566100	3.14053600	-0.00522100
C	1.37677200	1.89782000	-0.00517000
C	2.83665700	-0.52674400	-0.00077100
C	-2.82707500	-0.57441500	-0.00057200
C	3.53504000	-0.51860400	-1.20828900
C	4.92637300	-0.51097300	-1.20650100
C	5.61543400	-0.50779200	0.00741900
C	4.91945900	-0.51681400	1.21708400
C	3.52796700	-0.52427000	1.21061700
C	-3.52526300	-0.58227600	-1.20818500
C	-4.91653300	-0.59832400	-1.20662900
C	-5.60573300	-0.60813700	0.00715200
C	-4.90996000	-0.59804900	1.21695000
C	-3.51855300	-0.58221600	1.21071400
C	7.11799100	-0.55163500	0.01016900
C	-7.10853900	-0.57361000	0.01001700
F	-7.64259900	-1.19514600	-1.06680800
F	7.65585900	0.07257500	-1.06322500
F	7.65150000	0.02606900	1.11093900
F	7.58955400	-1.82855400	-0.01656600
F	-7.63848400	-1.16075000	1.10757100
F	-7.58794500	0.70048400	-0.00954700
C	-1.43727600	4.36045300	-0.00566900
C	-0.75340800	5.55964500	-0.00563500
C	0.65801100	5.57178700	-0.00535500
C	1.36244600	4.38455100	-0.00512500
H	-1.20517500	-5.15007700	-0.01905100
H	1.29309100	-5.12872000	-0.02010300
H	-2.44128600	-3.02298000	-0.01185900
H	2.49273000	-2.98094200	-0.01364300
H	-2.49060800	1.87244100	-0.00550600
H	2.45840100	1.91474700	-0.00490700
H	2.98822400	-0.51713200	-2.14322100
H	5.46818100	-0.50203500	-2.14365700
H	5.45574100	-0.51247100	2.15735100
H	2.97569300	-0.52713800	2.14232600
H	-2.97835100	-0.57824500	-2.14305200
H	-5.45810700	-0.60895800	-2.14389100
H	-5.44631300	-0.60837000	2.15713900
H	-2.96644900	-0.57814500	2.14251900
H	-2.52253300	4.34979900	-0.00586200
H	-1.30009100	6.49598600	-0.00581300
H	1.18849300	6.51740200	-0.00531900
H	2.44771700	4.39266500	-0.00490900

Coordinates of $\mathbf{2}^{+\bullet}$:

1 2

C	-0.66874700	-4.18089800	-0.00026800
C	0.73713200	-4.16937700	-0.00029300
C	-1.37523300	-2.99690300	-0.00013900
C	-0.69817800	-1.76157400	-0.00003100
C	0.72721900	-1.74985500	-0.00003000
C	1.42427300	-2.97404200	-0.00017400
N	-1.38775900	-0.56169500	0.00010000
C	-0.72490800	0.66344600	0.00021200
C	0.71402300	0.67533300	0.00025600
N	1.39695800	-0.53876900	0.00013200
C	-1.41723700	1.86452100	0.00024000
C	-0.74358800	3.09793300	0.00032500
C	0.69259400	3.10982900	0.00040700
C	1.38655600	1.88763300	0.00037200
C	2.84663800	-0.52942300	0.00001800
C	-2.83741700	-0.57488800	0.00002800
C	3.52663000	-0.52315300	-1.21438700
C	4.91800300	-0.51308200	-1.21280400
C	5.60799400	-0.50583600	-0.00025800
C	4.91822200	-0.51317900	1.21245100
C	3.52687100	-0.52326500	1.21431400
C	-3.51747300	-0.58069400	-1.21435200
C	-4.90881500	-0.59310200	-1.21270500
C	-5.59875700	-0.60107500	-0.00012200
C	-4.90894100	-0.59309800	1.21254000
C	-3.51760300	-0.58068600	1.21433900
C	7.11408000	-0.54425200	-0.00024200
C	-7.10489400	-0.56479900	-0.00017800
F	-7.63225600	-1.16879400	-1.08762500
F	7.64219100	0.05732600	-1.08875400
F	7.64217100	0.06201600	1.08573000
F	7.58277400	-1.81921000	0.00255200
F	-7.63233800	-1.16950300	1.08684400
F	-7.57509800	0.70982200	0.00022600
C	-1.44460300	4.33689200	0.00031400
C	-0.75670200	5.52280400	0.00037800
C	0.66501700	5.53463400	0.00046700
C	1.37265500	4.36037500	0.00048500
H	-1.20401700	-5.12186200	-0.00034500
H	1.28777400	-5.10143000	-0.00039800
H	-2.45510700	-3.01125500	-0.00011100
H	2.50424800	-2.97098000	-0.00018200
H	-2.49786700	1.86028900	0.00018400
H	2.46715300	1.90098200	0.00041500
H	2.97616900	-0.52501500	-2.14677100
H	5.45687500	-0.50537400	-2.15116200
H	5.45728300	-0.50550200	2.15071200
H	2.97657900	-0.52519200	2.14680000
H	-2.96706100	-0.57776800	-2.14676000
H	-5.44770100	-0.60221100	-2.15104700
H	-5.44793000	-0.60221300	2.15082500
H	-2.96728700	-0.57775800	2.14680300
H	-2.52861900	4.32731600	0.00024700
H	-1.29594900	6.46260900	0.00036000
H	1.18857100	6.48327500	0.00051700
H	2.45663400	4.36910900	0.00054900

Coordinates of **3**:

0 1

C	0.70642800	6.12595600	-0.01975900
C	-0.70642600	6.12595600	-0.01976300
C	-1.40063000	4.93422400	-0.01672200
C	-0.71279500	3.69487300	-0.01367700
C	0.71279600	3.69487200	-0.01367500
C	1.40063100	4.93422400	-0.01671600
C	-1.39310200	2.44863600	-0.01078000
C	-0.71819000	1.24631000	-0.00806800
C	0.71819100	1.24630900	-0.00806700
C	1.39310300	2.44863600	-0.01077600
N	-1.39558300	0.01583700	-0.00417800
C	-0.71817100	-1.21473600	-0.00829500
C	0.71817100	-1.21473600	-0.00829500
N	1.39558300	0.01583600	-0.00417700
C	-1.39308900	-2.41706200	-0.01131200
C	-0.71278900	-3.66328600	-0.01435300
C	0.71278800	-3.66328600	-0.01435400
C	1.39308800	-2.41706200	-0.01131300
C	-1.40065200	-4.90263100	-0.01750000
C	-0.70643500	-6.09434900	-0.02061600
C	0.70643300	-6.09434900	-0.02061800
C	1.40065000	-4.90263100	-0.01750300
C	2.83199100	0.01545000	0.00175300
C	-2.83199100	0.01545100	0.00175200
C	3.53095000	0.01713400	-1.20503900
C	4.92226500	0.01727900	-1.20065900
C	5.60914400	0.01843200	0.01453100
C	4.91137200	0.01510000	1.22313000
C	3.51985200	0.01516300	1.21461600
C	-3.51985100	0.01516900	1.21461500
C	-4.91137200	0.01510600	1.22312900
C	-5.60914400	0.01843300	0.01453100
C	-4.92226500	0.01727400	-1.20065900
C	-3.53095000	0.01713000	-1.20504000
C	7.11164500	-0.03275200	0.01929800
F	7.65374800	0.59461800	-1.05010000
C	-7.11164500	-0.03275200	0.01929900
F	-7.64618800	0.53651700	1.12377100
F	-7.65374800	0.59461400	-1.05010200
F	-7.57670900	-1.31173700	-0.01375900
F	7.64618900	0.53651300	1.12377200
F	7.57670900	-1.31173800	-0.01376400
H	1.24433600	7.06732000	-0.02215500
H	-1.24433400	7.06732000	-0.02216100
H	-2.48587400	4.93295600	-0.01673100
H	2.48587600	4.93295600	-0.01671900
H	-2.47472000	2.45610200	-0.01050000
H	2.47472100	2.45610100	-0.01049300
H	-2.47469600	-2.42453000	-0.01111600
H	2.47469500	-2.42453100	-0.01111900
H	-2.48589300	-4.90133900	-0.01749800
H	-1.24434100	-7.03571500	-0.02306100
H	1.24433900	-7.03571500	-0.02306400
H	2.48589200	-4.90134000	-0.01750300
H	2.98548200	0.02015900	-2.14071200
H	5.46569300	0.02223800	-2.13688700
H	5.44614600	0.01827100	2.16420600

H	2.96580200	0.01669000	2.14522900
H	-2.96580100	0.01670000	2.14522800
H	-5.44614600	0.01828100	2.16420600
H	-5.46569400	0.02223000	-2.13688700
H	-2.98548200	0.02015100	-2.14071300

Coordinates of **3**$^{+\bullet}$:

1 2

C	0.71169000	6.08762600	-0.00005900
C	-0.71168400	6.08762700	-0.00008000
C	-1.40940100	4.90862200	-0.00008600
C	-0.71903300	3.66235400	-0.00007000
C	0.71903600	3.66235400	-0.00005000
C	1.40940600	4.90862100	-0.00004400
C	-1.40232700	2.43685500	-0.00007400
C	-0.72040200	1.22698400	-0.00006300
C	0.72040300	1.22698300	-0.00004600
C	1.40233000	2.43685300	-0.00003900
N	-1.39300300	0.01255300	-0.00007000
C	-0.72040900	-1.20189900	-0.00008300
C	0.72040700	-1.20190000	-0.00004900
N	1.39300300	0.01255200	-0.00003200
C	-1.40235400	-2.41175500	-0.00011300
C	-0.71904200	-3.63724500	-0.00010700
C	0.71903800	-3.63724600	-0.00006100
C	1.40235200	-2.41175600	-0.00003500
C	-1.40942200	-4.88350600	-0.00014200
C	-0.71169500	-6.06250600	-0.00012700
C	0.71168800	-6.06250700	-0.00007600
C	1.40941700	-4.88350700	-0.00004400
C	2.84239100	0.01242500	-0.00003100
C	-2.84239100	0.01242800	0.00001200
C	3.52257900	0.01369300	-1.21436600
C	4.91398300	0.01577000	-1.21269700
C	5.60391300	0.01923600	-0.00005200
C	4.91399100	0.01578300	1.21261600
C	3.52259700	0.01370200	1.21430700
C	-3.52249900	0.01367500	1.21438900
C	-4.91390500	0.01575000	1.21281400
C	-5.60391300	0.01923900	0.00021700
C	-4.91406900	0.01581000	-1.21249900
C	-3.52267700	0.01372700	-1.21428300
C	7.10973400	-0.02615400	0.00001400
F	7.64076000	0.57415000	-1.08781500
C	-7.10973400	-0.02615200	0.00021200
F	-7.64071200	0.57354700	1.08839200
F	-7.64072800	0.57701500	-1.08607900
F	-7.57284500	-1.30331200	-0.00184700
F	7.64067900	0.57640900	1.08665700
F	7.57284600	-1.30331500	0.00137500
H	1.24252100	7.03220900	-0.00005400
H	-1.24251300	7.03221000	-0.00009300
H	-2.49342800	4.90829100	-0.00010200
H	2.49343300	4.90828800	-0.00002800
H	-2.48287900	2.44176100	-0.00008900
H	2.48288200	2.44175900	-0.00002400
H	-2.48291200	-2.41657200	-0.00014000
H	2.48291000	-2.41657400	-0.00000600

H	-2.49344700	-4.88314500	-0.00018000
H	-1.24252700	-7.00708800	-0.00015400
H	1.24252000	-7.00708900	-0.00006200
H	2.49344200	-4.88314700	-0.00000600
H	2.97204500	0.01506000	-2.14671100
H	5.45302300	0.02065700	-2.15099200
H	5.45304700	0.02069600	2.15090800
H	2.97207100	0.01507900	2.14665700
H	-2.97190300	0.01502600	2.14669800
H	-5.45288200	0.02061600	2.15114400
H	-5.45318800	0.02074600	-2.15075600
H	-2.97221200	0.01512000	-2.14667000

S12. References

[1] G. R. Fulmer, A. J. Miller, N. H. Sherden, H. E. Gottlieb, A. Nudelman, B. M. Stoltz, J. E. Bercaw, K. I. Goldberg, *Organometallics*, 2010, **29**, 2176-2179.

[2] T. M. Krygowski, M. Cyrański, *Tetrahedron*, 1996, **52**, 10255-10264.

[3] Gaussian 16, Revision B.01, M. J. Frisch, G. W. Trucks, H. B. Schlegel, G. E. Scuseria, M. A. Robb, J. R. Cheeseman, G. Scalmani, V. Barone, G. A. Petersson, H. Nakatsuji, X. Li, M. Caricato, A. V. Marenich, J. Bloino, B. G. Janesko, R. Gomperts, B. Mennucci, H. P. Hratchian, J. V. Ortiz, A. F. Izmaylov, J. L. Sonnenberg, D. Williams-Young, F. Ding, F. Lipparini, F. Egidi, J. Goings, B. Peng, A. Petrone, T. Henderson, D. Ranasinghe, V. G. Zakrzewski, J. Gao, N. Rega, G. Zheng, W. Liang, M. Hada, M. Ehara, K. Toyota, R. Fukuda, J. Hasegawa, M. Ishida, T. Nakajima, Y. Honda, O. Kitao, H. Nakai, T. Vreven, K. Throssell, J. A. Montgomery, Jr., J. E. Peralta, F. Ogliaro, M. J. Bearpark, J. J. Heyd, E. N. Brothers, K. N. Kudin, V. N. Staroverov, T. A. Keith, R. Kobayashi, J. Normand, K. Raghavachari, A. P. Rendell, J. C. Burant, S. S. Iyengar, J. Tomasi, M. Cossi, J. M. Millam, M. Klene, C. Adamo, R. Cammi, J. W. Ochterski, R. L. Martin, K. Morokuma, O. Farkas, J. B. Foresman, D. J. Fox, Gaussian, Inc., Wallingford CT, 2016.

[4] C. M. Cardona, W. Li, A. E. Kaifer, D. Stockdale, G. C. Bazan, *Adv. Mater.*, 2011, **23**, 2367-2371.

[5] J. P. Cole, C. R. Federico, C.-H. Lim, G. M. Miyake, *Macromolecules*, **2019**, *52*, 747-754.
